# Molecular Characterization and Seroprevalence of Hepatitis E Virus in Inflammatory Bowel Disease Patients and Solid Organ Transplant Recipients

**DOI:** 10.3390/v13040670

**Published:** 2021-04-13

**Authors:** Juozas Grigas, Maria Montoya, Evelina Simkute, Marius Buitkus, Ruta Zagrabskaite, Arnoldas Pautienius, Dainius Razukevicius, Laimas Virginijus Jonaitis, Gediminas Kiudelis, Jurgita Skieceviciene, Ruta Vaiciuniene, Asta Stankuviene, Inga Arune Bumblyte, Juozas Kupcinskas, Arunas Stankevicius

**Affiliations:** 1 Laboratory of Immunology, Department of Anatomy and Physiology, Lithuanian University of Health Sciences, Tilzes Str. 18, LT-47181 Kaunas, Lithuania; evelina.simkute@lsmu.lt (E.S.); arnoldas.pautienius@lsmuni.lt (A.P.); arunas.stankevicius@lsmuni.lt (A.S.); 2 Institute of Microbiology and Virology, Lithuanian University of Health Sciences, Tilzes Str. 18, LT-47181 Kaunas, Lithuania; 3 Centro de Investigaciones Biológicas Margarita Salas, Consejo Superior de Investigaciones Cientificas (CIB-CSIC), Calle Ramiro de Maeztu, 9, 28040 Madrid, Spain; maria.montoya@cib.csic.es; 4 Institute of Clinical Medicine, Vilnius University, M. K. Ciurlionio Str. 21/27, LT-03101 Vilnius, Lithuania; marius.buitkus@vrm.lt; 5 Serology Unit, National Food and Veterinary Risk Assessment Institute, J. Kairiukscio Str. 10, LT-08409 Vilnius, Lithuania; ruta.zagrabskaite@nmvrvi.lt; 6 Department of Maxillofacial Surgery, Faculty of Odontology, Lithuanian University of Health Sciences, A. Mickeviciaus Str. 9, LT-44307 Kaunas, Lithuania; dainius.razukevicius@lsmu.lt; 7 Department of Gastroenterology, Faculty of Medicine, Lithuanian University of Health Sciences, Eiveniu Str. 2, LT-50161 Kaunas, Lithuania; laimas.jonaitis@lsmuni.lt (L.V.J.); gediminas.kiudelis@lsmuni.lt (G.K.); jurgita.skieceviciene@lsmuni.lt (J.S.); juozas.kupcinskas@lsmuni.lt (J.K.); 8 Institute of Digestive Research, Faculty of Medicine, Lithuanian University of Health Sciences, Eiveniu Str. 2, LT-50161 Kaunas, Lithuania; 9 Department of Nephrology, Faculty of Medicine, Lithuanian University of Health Sciences, Eiveniu Str. 2, LT-50161 Kaunas, Lithuania; ruta.vaiciuniene@lsmuni.lt (R.V.); asta.stankuviene@lsmu.lt (A.S.); ingaarune.bumblyte@lsmuni.lt (I.A.B.)

**Keywords:** hepatitis E, inflammatory bowel disease, biological therapy, solid organ transplant, immunosuppression, MARC-145, genotype 3, zoonosis, Crohn’s disease, ulcerative colitis

## Abstract

Seroprevalence rates and molecular characterization of hepatitis E virus (HEV) prevalent in the Lithuanian human population has not yet been evaluated. Immunosuppressed individuals have been recognized as a risk group for chronic hepatitis due to HEV genotype 3 (HEV-3) infections. The objectives of the present study were to determine prevalence rates of anti-HEV antibodies among inflammatory bowel disease (IBD) patients and solid organ transplant (SOT) recipients, to isolate and characterize HEV strain present in the Lithuanian human population, and to investigate its capacity to infect non-human primate (MARC-145 and Vero), swine (PK-15) and murine (Neuro-2a) cells in vitro. In the present study, the significant difference of anti-HEV IgG prevalence between healthy (3.0% (95% CI 0–6.3)) and immunosuppressed individuals (12.0% [95% CI 8.1–15.9]) was described. Moreover, our findings showed that anti-HEV IgG seropositivity can be significantly predicted by increasing age (OR = 1.032, *p* < 0.01), diagnosis of IBD (OR = 4.541, *p* < 0.01) and reception of SOT (OR = 4.042, <0.05). Locally isolated HEV strain clustered within genotype 3i subtype of genotype 3 and was capable of infecting MARC-145 cells. This study demonstrates higher HEV seroprevalence in the risk group compared to healthy control individuals without confidence interval overlap. The high level of genetic homology between human and animal strains in Lithuania and the capacity of locally isolated strains to infect cells of non-human origin suggests its potential for zoonotic transmission.

## 1. Introduction

Hepatitis E virus (HEV) is a causative agent of human and animal hepatitis E belonging to the genus *Orthohepevirus*. Species *Orthohepevirus A* consists of at least 7 HEV genotypes, 5 of which are associated with human infections and 3 of them (HEV-3, HEV-4, and HEV-7) are capable of zoonotic transfer [1]. HEV-3 and HEV-4 have mainly been associated with human and animal hepatitis E infections in industrialized countries in Europe and North America. The predominant transmission pathway of both HEV-3 and HEV-4 is considered to be zoonotic, with domestic pigs (*Sus scrofa domesticus*) and wild boars (*Sus scrofa*) serving as the most important reservoirs [2,3].

Crohn’s disease (CD) and ulcerative colitis (UC) both fall under an umbrella term of inflammatory bowel disease (IBD). The underlying mechanism of IBD has been associated with the defective response of the host immune system to gut microbiota [4]. Environmental factors like smoking, air pollution, and low vitamin D levels have also been shown to increase both incidence rates and disease progression of IBD [5]. In addition, some viral infections have been associated with exacerbation of IBD [6,7], however, there is no concluding evidence supporting their role in IBD etiopathogenesis. Although IBD patients have previously been considered a risk group for hepatitis B (HBV) and C (HCV) [7], recent studies demonstrated that prevalence rates of viral hepatitis differ little between IBD patients and the general population [8,9]. Hepatitis E rarely manifests clinical signs in immunocompetent individuals, while patients receiving immunosuppressive therapy are more susceptible to the chronic course of HEV [10]. Immunomodulatory drugs and biological therapy have been extensively used to control IBD, leaving the patient vulnerable to opportunistic pathogens [11]. Despite the lack of data supporting the causal links between viral hepatitis and IBD per se, immunomodulatory treatment of IBD might leave patients more susceptible to viral infections. 

Among the immunosuppressed individuals, blood transfusion and solid organ transplant (SOT) recipients, and especially liver allograft recipients, have been recognized as risk groups for the development of chronic HEV-3 [12,13]. In a recent study, an HEV-3 seroprevalence rate of 55.6% was observed in liver allograft recipients, with an active hepatitis rate of 2.8% [13]. This is a significantly higher HEV-3 seroprevalence rate compared to the general population, where anti-HEV antibody-positive individual rates range from 0.1% to 12% on average [14]. Occult HEV carriers that are seronegative for anti-HEV antibodies, but are nonetheless infected by HEV, have been of particular interest, especially in the cases of organ transplantation. Cases of hepatitis E in liver allograft recipients from anti-HEV antibody and serum HEV RNA-free donors, that later had HEV RNA detected in liver samples, have been described [15]. Moreover, wild boar studies have demonstrated that a significant proportion of animals with HEV viremia did not exhibit seropositivity for anti-HEV antibodies [16]. Considering the high seroprevalence rate of liver allograft recipients and the dangers of occult HEV carriers as donors, correct detection of subclinical HEV infections is crucial for avoiding HEV-related complications of the post-SOT period.

To date, no studies evaluating anti-HEV antibody seroprevalence and direct serum HEV RNA rates in the Lithuanian human population have been carried out. In our previous study, we have demonstrated seroprevalence rates of 43.8% and 57.1% in Lithuanian domestic pig and wild boar populations, respectively, which are considered to be important reservoirs for HEV-3 [17]. Both widespread pork consumption and hunting practices in Lithuania are similar to its neighboring countries. Therefore, we expect similar HEV seroprevalence rates in risk groups and the general population to other Eastern European countries [18,19,20].

Both prevalence and genetic data of HEV in Lithuania are scarce. Moreover, no studies investigating link between IBD and HEV attempted to assess the risk by comparing HEV prevalence data in healthy controls and IBD patients. Therefore, the objectives of our study were: (1) to determine HEV prevalence rates in human risk group cohorts; (2) to isolate, characterize and compare local human HEV strains with animal strains; (3) to evaluate the risk of HEV in immunosuppressed patient groups.

In this study, anti-HEV IgG and IgM seroprevalence and serum HEV RNA rates in healthy control, IBD patients, and SOT recipients of Lithuania were demonstrated. Moreover, phylogenetic analysis and genetic comparison between isolates from human and animal populations of Lithuania were carried out. Finally, a local HEV strain was used to experimentally infect kidney and neuronal cell lines in vitro.

## 2. Materials and Methods

### 2.1. Serum Samples

Serum samples (*n* = 366) were collected from:

1. Patients hospitalized in Hospital of Lithuanian University Health Sciences Kaunas Clinics (HLUHSKC) Gastroenterology clinic and diagnosed with IBD (*n* = 203). All samples were assigned into 2 groups based on clinical characteristics, namely patients with diagnosed UC (*n* = 156) or CD (*n* = 47). In addition, all IBD patients were assigned into groups based on whether they have (*n* = 42) or have not (*n* = 161) received biological therapy (anti-tumor necrosis factor (TNF) (infliximab/adalimumab) antibody protocol). Samples from anti-TNF therapy group patients were collected during the maintenance stage of the treatment. Samples were collected from 2015 to 2020, stored, and retrospectively tested for the presence of anti-HEV antibodies and HEV RNA.
2. SOT recipients were hospitalized in HLUHSKC Nephrology clinic and Gastroenterology clinic (*n* = 63). Samples were collected during post-transplantation visitations. All samples were assigned into 2 groups based on allograft type, namely kidney (*n* = 58) and liver (*n* = 5). Samples were collected from 2019 to 2020 and tested immediately after collection.
3. Healthy control group (*n* = 100) of individuals without clinical IBD diagnosis or SOT. Sufficient representation of different age groups and genders was achieved by the inclusion of an equal number of samples from each category. Samples were collected from 2015 to 2017, stored, and retrospectively tested for the presence of anti-HEV antibodies and HEV RNA.

Part of the patient and control samples included in this study came from previously studied cohorts of patients with IBD [21,22,23] and liver disease [24,25,26,27]. Sample data is presented in Table 1. All samples were stored at −80 °C before testing.

### 2.2. Serological Testing

All collected samples were tested by ELISA for the presence of anti-HEV IgG and IgM antibodies, using commercially available kits (recomWell HEV IgG or IgM, Mikrogen GmbH, Neuried, Germany) according to the manufacturer’s instructions. All borderline samples were retested and confirmed as either positive or negative. Anti-HEV IgG-positive samples represented lifetime exposure, while anti-HEV IgM-positive (and either IgG-positive or negative) were classified as acute infections.

### 2.3. Molecular Diagnostics

Total RNA was extracted from serum samples using a commercially available GeneJET RNA Purification Kit (Thermo Scientific, Waltham, MA, USA) according to the manufacturer’s instructions. Reverse transcription-polymerase chain reaction (RT-PCR) was carried out targeting ORF1 and ORF2 regions of HEV RNA and performed with all samples regardless of ELISA results using primer sets and cycling conditions described previously [28]. For cDNA synthesis, RevertAid reverse transcriptase (Thermo Scientific, Waltham, MA, USA) and RiboLock RNase inhibitor (Thermo Scientific, Waltham, MA, USA) were used. DreamTaq Green PCR Master Mix (2×) (Thermo Scientific, Waltham, MA, USA) was used for both cDNA synthesis and PCR. All samples were tested in triplicates.

### 2.4. Sequencing and Phylogenetic Analysis

Further genetic characterization of human-derived HEV isolates partial genome sequencing was performed, using a previously described primer set [29]. The resulting partial ORF2 sequence was submitted to GenBank (Accession number MT585816). Phylogenetic analysis was carried out by comparing local human-derived strains to HEV-3 reference strains isolated in human, swine, or wild boar hosts. Multiple alignments were created using ClustalW software in the MEGA X package. The neighbor-joining tree was constructed with bootstrap testing across 1000 replicates.

### 2.5. Viral Stock Preparation and Cell Line Infection

The viral stock was prepared from the serum of ORF1/ORF2-positive patients (HEV strain MT585816). The sample was diluted in PBS (1×, pH 7.2) (Gibco, Grand Island, NY, USA) and centrifuged at 3000× *g* and 12,000× *g* for 10 and 5 min, respectively, until a supernatant was acquired and passed through a 0.22 μm pore size microfilter (TPP Techno Plastic Products AG, Trasadingen, Switzerland) for purification. HEV RNA load was determined to be 2.5 × 107 copies/mL as previously described [30].

Monkey kidney cells (MARC-145 ATCC No. CRL-12231; Vero ATCC No. CCL-81) (ATCC^®^, Manassas, VA, USA), porcine kidney cells (PK-15 ATCC No. CCL-33), and murine neuroblastoma cells (Neuro-2a ATCC No. CCL-131) were used for experimental infection with HEV viral stock. Cell lines were cultured in previously described conditions [30]. Cells were trypsinized following the formation of the monolayer, diluted (1:3 and 1:6 dilutions for Neuro-2a and PK-15 cells respectively, and 1:4 for MARC-145 and Vero cell lines) in Minimum Essential Medium (MEM; Gibco, Grand Island, NY, USA) (MARC-145, Vero and Neuro-2a cells) or Dulbecco’s modified Eagle’s medium (DMEM; Gibco, Grand Island, NY, USA) (Neuro-2a cells) with 10% heat-inactivated fetal bovine serum (FBS; Gibco, Grand Island, NY, USA), 100 U/mL penicillin and 100 μg/mL streptomycin, and transferred to 10 cm^2^ tissue culture flasks (TTP Techno Plastic Products TP, Trasadingen, Switzerland) 1 day before HEV inoculation.

Experimental infection was carried out by removing the growth medium from the flask, washing with 5 mL PBS and inoculating cell monolayer with 1 mL purified virus stock. The infection mixture was removed after 1 h of incubation at room temperature and replaced with 5 mL of maintenance medium (DMEM for Neuro-2a and MEM for other cells with the above-described additives). HEV-exposed cells were cultured for 14 days at 37 °C, replacing 2.5 mL of maintenance medium every 2 to 3 days. The presence of HEV RNA in the maintenance medium was tested at 14 dpi in all cell lines using RT-PCR as described above. All infections were performed in duplicate sets.

### 2.6. Immunofluorescence Assay and Confocal Microscopy

At 14 dpi, flasks with HEV-exposed cells were emptied of maintenance medium, washed with PBS, and fixed using ice-cold methanol (Carl Roth Gmbh, Karlsruhe, Germany) and acetone (Carl Roth Gmbh, Karlsruhe, Germany) mixture in 1:1 ratio at 1 mL/slide for 5 min at −20 °C. Slides were air-dried and stored at 4 °C until further use. HEV capsid protein presence in infected cell cultures was confirmed by immunofluorescence assay, using HEV-3 capsid protein-specific monoclonal antibody clone 5F3 (Alexa Fluor 488; mAb 5F3) [31], Phalloidin (FITC; F432) (Life Technologies, Carlsbad, CA, USA) and Diamond Antifade Mountant with 4′,6-diamidino-2-phenylindole (DAPI) (Invitrogen, Carlsbad, CA, USA) for HEV capsid protein, cytoskeletal and nuclei staining respectively. Fluorescence microscopy was performed using Zeiss LSM 700 (Carl Zeiss AG, Jena, Germany) confocal microscope. Images were acquired using Plan-Apochromat 40 × 0.8 oil DIC M27, while z-stacks were captured every 1 μm and analyzed using ImageJ software.

### 2.7. Statistical Analysis

Seroprevalence data were presented in percentages with 95% confidence intervals (CI). Continuous data were presented as medians with a standard deviation (SD). Risk assessment of different factors was examined using univariate logistic regression analysis and expressed as odds ratios (OR) with 95% CI. *p* < 0.05 was considered statistically significant.

## 3. Results

### 3.1. HEV Seroprevalence in IBD Patients and SOT Recipients

**Figure 1:**
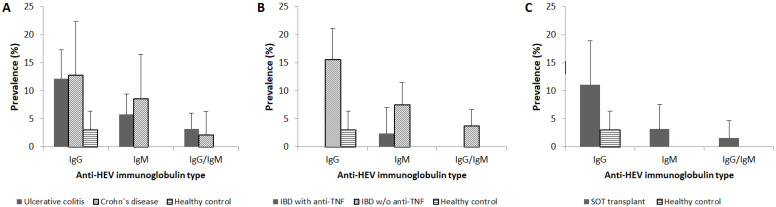
Seroprevalence of HEV in (**A**) IBD types, (**B**) IBD patients with or without anti-TNF treatment, and (**C**) SOT recipients, compared to the healthy control. IgG—immunoglobulin G; IgM—immunoglobulin M; w/o—without.

Out of 366 tested individuals, 9.6% (95% CI 6.6–12.6) were anti-HEV IgG positive, 4.1% (95% CI 2.1–6.1) were anti-HEV IgM positive, and 1.9% (95% CI 0.5–3.3) were anti-HEV IgG/IgM positive. Out of 100 healthy control group samples, 3.0% (95% CI 0–6.3) were anti-HEV IgG positive, whereas 12.0% (95% CI 8.1–15.9) of individuals with IBD or SOT were anti-HEV IgG positive, with no confidence interval overlap (Table 2). No anti-HEV IgM-positive samples were found among the healthy control (Figure 1). HEV seroprevalence rates in age groups of years 30–39, 50–59, and 60–69 were higher among risk group individuals with anti-HEV IgG prevalence rates of 18.0% (95% CI 7.4–28.7), 13.3% (95% CI 3.4–23.3) and 29.0% (95% CI 13.1–45.0) respectively, compared to healthy control with no confidence interval overlap (Table 2). HEV seroprevalence in both genders was also higher among risk group individuals. All positive anti-HEV IgG samples in the healthy control group were detected among male individuals.

Among patients with IBD, 12.3% (95% CI 7.8–16.8) were anti-HEV IgG positive, compared to 3.0% (95% CI 0–6.3) detected in healthy control with no confidence interval overlap (Table 3). 6.4% (95% CI 3.0–9.8) and 3.0% (95% CI 0.6–5.3) of IBD patients were anti-HEV IgM and IgG/IgM positive, respectively. Higher anti-HEV IgG and IgM prevalence rates were found among CD (12.8% (95% CI 3.2–22.3) and 8.5% (95% CI 0.5–16.5) for IgGs and IgMs, respectively) patients, compared to the UC group (12.2% (95% CI 7.1–17.3) and 5.8% (95% CI 2.1–9.4) for IgGs and IgMs, respectively (Figure 1A). Anti-HEV IgG, IgM, and IgG/IgM prevalence rates were higher among IBD patients that did not receive anti-TNF treatment (15.5% (95% CI 9.9–21.1), 7.5% (95% CI 3.4–11.5) and 3.7% (95% CI 0.8–6.7) for IgGs, IgMs, and IgGs/IgMs, respectively), compared to IBD patients that did and healthy control group (Figure 1B). In the UC group, the highest anti-HEV IgG, IgM, and IgG/IgM prevalence rates were detected among patients aged 60–69 (50.0 (95% CI 23.8–76.2), 14.3 (95% CI 0–32.6) and 14.3 (95% CI 0–32.6) for IgGs, IgMs and IgGs/IgMs, respectively), while no anti-HEV IgGs were detected in UC patient group aged 50–59 (Table 3). Among CD patients, no clear statistical difference in seroprevalence between age groups was observed, with anti-HEV IgGs detected in patients aged 30–39, 50–59, and 60–69, and anti-HEV IgMs detected in patients aged ≤29 and 30–39 (Table 3). Among IBD patients that received anti-TNF treatment, only one 57-year-old individual was anti-HEV IgM positive, while none of the tested sera was anti-HEV IgG-positive (Figure 1B).

Among SOT recipients, 11.1% (95% CI 3.4–18.9) were anti-HEV IgG positive, compared to 3.0% (95% CI 0–6.3) in the healthy control group with confidence interval overlap (Figure 1C). 3.2% (95% CI 0–7.5) and 1.6% (95% CI 0–4.7) of SOT recipients were anti-HEV IgM and IgG/IgM positive, respectively. Although a higher anti-HEV IgG prevalence rate was detected in the liver allograft recipient group (20.0% (95% CI 0–55.1)) compared to the kidney allograft recipient group (10.4% (95% CI 2.5–18.2)) and healthy control, a statistically significant difference was not achieved, probably due to low sample size of liver allograft recipient group. In SOT recipients, anti-HEV IgG-positive individuals clustered within 40–49 and 50–59-year age groups (11.1% (95% CI 0–31.6) and 26.3% (95% CI 6.5–16.1), respectively) with an overlap in confidence intervals, while anti-HEV IgM-positive individuals clustered within 50–59 and 60–69-year age groups (5.3% (95% CI 0–15.3) and 7.1% (95% CI 0–20.6), respectively) (Table 3). A single anti-HEV IgG-positive individual with liver allograft was an anti-HEV IgM-negative 54 years old male.

The logistic regression analysis (Table 4) demonstrated that anti-HEV IgG seropositivity can be significantly predicted by increasing age (OR = 1.032 (95% CI 1.007–1.057)), diagnosis of IBD (OR = 4.541 (95 % CI 1.337–15.426)) and both UC (OR = 5.688 (95% CI 1.633–19.817)) and CD (OR = 6.929 (95% CI 1.628–29.487)), and reception of SOT (OR = 4.042 (95% CI 1.005–16.258)) in a form of kidney (OR = 4.222 (95% CI 0.979–18.213)) or liver (OR = 12.432 (95% CI 0.992–155.757)) allograft. No significant association was demonstrated between anti-HEV IgG seropositivity and anti-TNF treatment or gender. Moreover, logistic regression analysis did not show any significant predictors for anti-HEV IgM or IgG/IgM seropositivity (data not shown).

### 3.2. RNA Detection and Molecular Characteristics of HEV in Lithuanian IBD Patients and SOT Recipients

All patient and healthy control blood sera samples were tested for the presence of HEV RNA using ORF1 and ORF2 as target sequences. All individuals that tested positive for HEV RNA are presented in Table 5. 2 patients (BT1 and KA1; Table 5) tested ORF2 positive and 4 patients (BT1, KA2, KA3, and KA4; Table 3) tested ORF1 positive. The majority of HEV RNA-positive individuals were kidney allograft recipients. Only one serum sample (BT1; Table 5) tested positive for both ORF1 and ORF2 and was used for viral stock preparation. Interestingly, only one sample (KA2; Table 5) was HEV RNA and anti-HEV Ig positive. The rest of HEV RNA-positive individuals were both anti-HEV IgG and IgM-negative.

**Figure 2:**
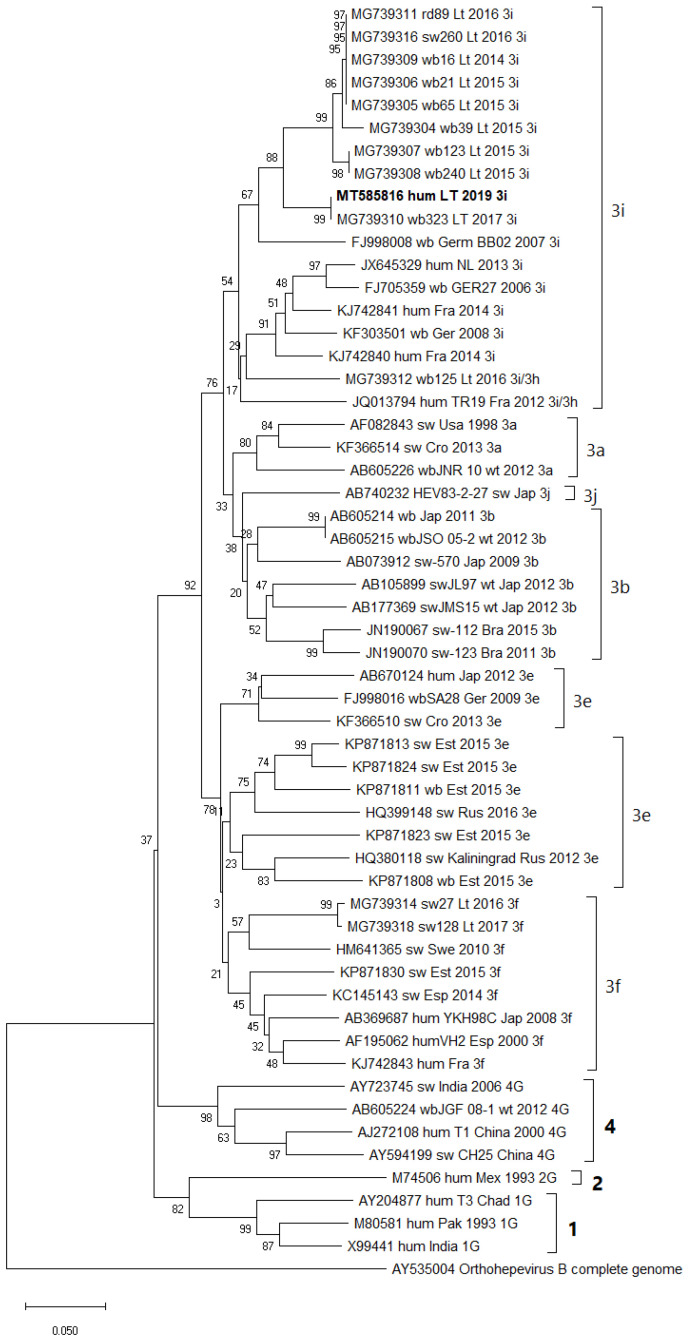
Phylogenetic analysis of partial HEV RNA sequence MT585816 from the human sample (highlighted in bold). Avian HEV (AY535004) was used as an outgroup.

Viral RNA from a serum sample of patient BT1 was successfully isolated and 243 nt long partial HEV ORF2 sequence was acquired (Accession number MT585816). Phylogenetic analysis showed that partial HEV RNA sequence belonged to genotype 3 and clustered within 3i subtype (Figure 2), together with HEV strains isolated from humans in France and the Netherlands, and fell within a well-supported lineage of other HEV 3i subtype isolates of wild boar and swine origin from Lithuania as previously described [28], sharing up to 99% homology with nearest strains.

### 3.3. HEV Isolate Propagation in MARC-145 Cells

**Figure 3:**
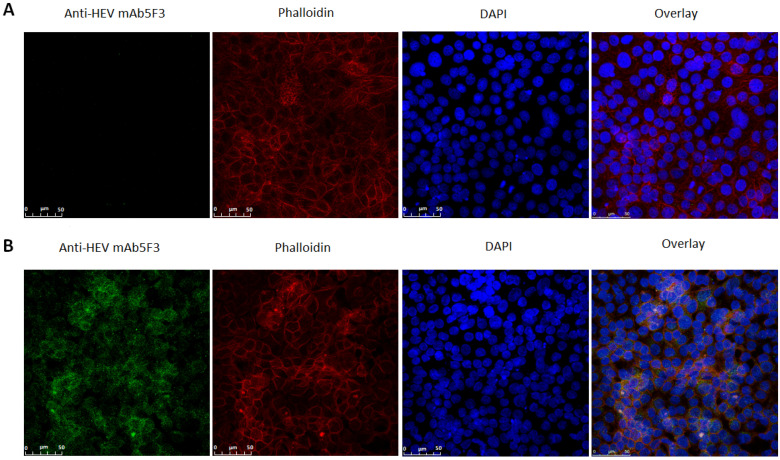
Confocal microscopy panels of uninfected (**A**) and HEV-infected (**B**) MARC-145 cells. mAb 5F3 (Alexa Fluor 488; green) were used for HEV capsid protein staining, phalloidin (red) for cytoskeletal staining and DAPI (blue) for cell nuclei staining. Scale bar: 50 μm.

To test the infectivity of locally isolated HEV strain from ORF1/ORF2-positive individuals (BT1; Table 5), experimental infections of MARC-145, Vero, PK-15, and Neuro-2a cell lines were carried out. Purified virus stock of BT1-derived HEV strain prepared from the serum sample was used for in vitro infection. Infected Vero, PK-15 and Neuro-2a cells tested negative for both HEV progeny RNA in maintenance medium and HEV capsid protein presence in the cytoplasm (data not shown). MARC-145 cell maintenance medium tested HEV RNA-positive at termination day (14 dpi) and cells were fixed for immunofluorescence assay and confocal microscopy. Fluorescent confocal microscopy confirmed the capacity of the MT585816 strain to be successfully presented in MARC-145 cells as seen in Figure 3B compared to the negative control (Figure 3A. Granular-like staining in infected cells can be seen distributed throughout the cytoplasm of infected cell groups, indicating viral RNA synthesis and subsequent HEV capsid protein translation from a sub-genomic viral mRNA. Cytoplasmic localization of viral particles was confirmed by multiple cross-sectional imaging at different monolayer height dimensions, enabling us to reject the possibility of viral particle adsorption on cell membrane surface without internalization (Figure S1). 

## 4. Discussion

To date, almost no information regarding HEV seroprevalence and genetic diversity in the Lithuanian population exists. The results presented here demonstrate a significantly higher risk of anti-HEV IgG presence among IBD patients and SOT recipients, compared to the healthy sample group. This is consistent with findings reporting increased risk of HEV infection among liver allograft recipients and increased risk of opportunistic pathogens in immunosuppressed individuals [11,13]. A substantial amount of research has been carried out investigating the relationship between IBD and different viral hepatitis forms (mainly HBV and HCV). However, only two studies that investigate associations between IBD and HEV have been published to date, demonstrating prevalence rates of HEV in IBD patients without comparing them to a random sample [11,19]. Therefore, our study is the first one attempting to investigate a significant correlation between HEV seroprevalence and IBD. The overall prevalence rate of anti-HEV IgG and IgM among IBD patients (12.3% and 6.4%, respectively) in our study was higher compared to similar studies (1.3% and 2.7% for IgG and IgM, respectively) [19]. Moreover, similarly to studies investigating the association between IBD and other hepatitis viruses [9], a comparison between seroprevalence data from IBD patients and healthy control allowed us to establish a significant link between CD/UC and prevalence of anti-HEV IgG. Interestingly, odds ratios for the presence of anti-HEV IgG and diagnosis of IBD were almost two times higher in our study (OR = 4.042) compared to links between IBD and HBV or HCV (OR = 2.5 and insignificant odds ratio, respectively) reported in other studies [9]. This suggests higher vulnerability among IBD patients to HEV infection, compared to HBV or HCV infections. This difference in odds ratio might be associated with differences in treatment, disease severity, or geographic variation of hepatitis virus strains. Therefore, a comparative study, evaluating the risk of viral hepatitis (including HEV) infection in the IBD patient sample would be necessary for confirmation.

Despite the lack of data reporting the impact of IBD patients receiving biological therapy have on HEV infection, a question of HBV reactivation in IBD patients receiving biological therapy and the potential impact of immunosuppression has been widely discussed without a clear consensus [32,33,34]. Our data show no significant change in HEV antibody seroprevalence in patients receiving anti-TNF therapy compared to healthy control. Interestingly, the only HEV RNA-positive patient with respect to both ORF1 and ORF2 genetic markers belonged to the group treated with anti-TNF antibodies (BT1; Table 5), who was also both anti-HEV IgG and IgM-negative. This is consistent with previously described recommendations to test all IBD patients and allograft recipients for HEV RNA, whether or not they are anti-HEV Ig-positive, because of a potential impact immunosuppressive therapy may have on antibody production mechanism [35]. Although changes in the immune system associated with IBD include an increase in both mucosal plasma cell population and levels of IgG, to date, biological therapy strategies have mostly been aimed at controlling innate immune response and cell-mediated immunity, since B cell and immunoglobulin role in IBD has not been fully understood [36]. Moreover, IBD treatment strategies associated with B cell depletion revealed counterproductive results, demonstrating exacerbation of UC after rituximab therapy [37]. Whatever the role of antibody-mediated immunity in IBD is, whether it is anti- or proinflammatory, the impact of different immunosuppressive biologics on anti-viral antibody production has not been investigated in detail. We speculate that the neutralization of important players in IBD pathogenesis, such as TNF, by infliximab or adalimumab, which in turn induce T cell apoptosis, may result in suppression of B cell co-stimulation pathway and inability to mount a sufficient antibody response to a viral antigen [38]. This would explain not only the absence of anti-HEV antibodies and presence of HEV RNA in patient BT1 but also the presence of HEV RNA in the serum sample, usually associated with viremia that in immunosuppressed individuals lasts for at least three months [39]. Testing the latter hypothesis would require a larger sample size of patients receiving anti-TNF treatment as the sample size used in the present study was not robust enough to show a statistically significant difference between the anti-TNF therapy group and the healthy control group.

The prevalence of HEV in SOT recipients has been widely studied in Europe and Japan, primarily in liver, kidney, and heart allograft recipients [35,40,41]. Among kidney allograft recipients in Lithuania, we report an anti-HEV IgG prevalence rate of 10.4%, which is similar to prevalence rates demonstrated in other European countries (mean anti-HEV IgG 11.7%) [41] and significantly higher compared to the reported rate in Japan (4.1%) [35]. Our study also demonstrates an elevated risk of anti-HEV IgG presence in kidney allograft recipients (OR = 4.222) compared to healthy control, consistent with previous findings from other European countries [42,43]. A number of anti-HEV IgM-positive and HEV RNA-positive individuals in the SOT recipient group was too low to establish a statistically significant difference. Despite a low sample size of liver allograft recipients, we also demonstrated a significant risk of anti-HEV IgG presence in the liver allograft recipient group (OR = 12.432; *p* = 0.05), however, the confidence interval range (95% CI 0.992–155.757) prevents us from concluding that the relationship between risk of HEV infection and liver transplantation exists. The higher anti-HEV IgG prevalence rate in liver allograft recipients (20.0%), compared to rates presented in similar papers [44,45,46], may also be skewed due to the relatively small sample size.

Upon examination of other possible risk factors, we have identified a slightly higher risk of anti-HEV IgG presence associated with age increase (OR = 1.032; *p* = 0.01), which is in agreement with other studies [47,48,49]. Interestingly, in both UC and CD patients, the highest anti-HEV IgG (and IgM in UC patients) prevalence rates were observed in individuals of ages 30–39 and 60–69 years. This trend is not reflected in the general population examined in our study and other papers [48,50]. Similar two-phase distribution is characteristic of adult UC onset, the incidence of which first peaks at age of 30–40 years, and then at age of 60–69 years [51]. This might suggest a pathogenetic link between the onset of UC and HEV infection. A similar age distribution is not characteristic of CD, where more than 80% of patients are diagnosed before the age of 40 [52]. This trend is reflected by the seroprevalence of anti-HEV IgMs, which were only detected in patients of age ≤29 and 30–39 groups (Table 3). To date, data from studies investigating gender as a risk factor for HEV infection have been inconclusive: both male [53] and female [48] gender has been identified as a potential risk factor for anti-HEV Ig presence. The majority of studies did not find any statistically significant link between gender and HEV seroprevalence [47,49]. Similarly, we did not find any differences in risk of anti-HEV IgG, IgM, or IgG/IgM presence between genders (Table 3).

HEV infection mixture prepared from a serum sample of patient BT1 was used for inoculation of MARC-145, PK-15, VERO, and Neuro-2a cell lines. In our previous findings, we demonstrated that HEV isolates from Lithuanian wild boar liver sample was capable of replication in all of the aforementioned cell lines [30]. In the present study, only MARC-145 cells were observed to successfully harbor HEV particles within the cytoplasm at 14 dpi. This difference in the viability of cell lines to allow for propagation of genetically similar HEV strains isolated from serum, as opposed to the liver sample, may be explained by the fact that HEV located in the serum retain quasi-enveloped configuration, which is characterized by slower entry compared to nonenveloped HEV usually found in the liver [54]. Although the aim of human HEV strain isolation was aimed at comparing its kinetics in cell cultures to similar Lithuanian HEV strains from wild animals, the present study supplements our earlier findings and suggests differences of MARC-145, PK-15, VERO, and Neuro-2a cell lines in their capacity to retain quasi-enveloped and nonenveloped HEV and facilitate its successful replication.

Phylogenetic analysis of a locally acquired HEV strain from a BT1 patient confirmed its position within genotype 3, consistent with other studies of autochthonous HEV strains in Europe [55,56]. Similar to our previous study of HEV genetic diversity in Lithuanian wild animals and pigs [28], the present study revealed clustering of HEV isolate MT585816 within the 3i subtype. It shares high homology with sequences of autochthonous Lithuanian strains from animals (particularly MG739310, wild boar-origin), wild boar strains from Germany (FJ998008), and human strains from the Netherlands and France (JX645329 and KJ42841, respectively). Zoonotic transmission pathway being the predominant one for HEV-3, genetic similarity between local human and animal strains suggests a link between human HEV cases and consumption of animal meat in Lithuania [2,57]. However, similarly to other Baltic and Eastern European countries, undercooked meat consumption is not culturally widespread in Lithuania. Potential HEV transmission routes other than consumption of animal meat had been analyzed by European research groups, suggesting a possibility of HEV transmission via professional or recreational exposure to wild boar or pigs [18,58,59]. Further studies, including a larger sample of autochthonous isolates from more diverse focus groups, would be necessary to investigate the relationship between animal and human transmission routes. Moreover, a comparison between capacities of HEV isolates from human and animal hosts to infect different cell lines in vitro may be employed to further supplement genetic data. Our findings revealed that HEV viral stocks prepared from wild boar [28] and human samples were both capable of successfully infecting MARC-145 cells. Possible differences of quasi-enveloped and nonenveloped HEV configurations in sera and liver samples may contribute to the inability of quasi-enveloped HEV to infect other cell lines that were permissive for nonenveloped HEV. Therefore, viral stock prepared from the same tissue source should be used to further investigate the capacity of Lithuanian strains to infect different cell lines regardless of the host.

Several limitations of the paper need to be addressed. First, all test group samples came from patients treated at HLUHSKC, which does not include samples from all demographic districts of Lithuania, underrepresenting agricultural regions of the country, where direct contact with wild animals and pigs is more common, depriving access to data of significant interest in terms of HEV epidemiology. Secondly, sample size within some patient groups (e.g., liver allograft recipient and IBD with anti-TNF treatment group) was not robust enough to conclude with confidence that increased risk of HEV infection is associated with an underlying medical condition. Thirdly, not enough IgM-positive and HEV RNA-positive samples were present in the study, preventing us from establishing significant relationships between anti-HEV IgM/HEV RNA presence and various risk factors. Lastly, HEV RNA of only one isolate was present in sufficient quantities in the serum sample for successful gene sequencing, in turn limiting the scope of molecular characterization and phylogenetic analysis of locally circulating HEV genetic variety in the Lithuanian human population.

## 5. Conclusions

In conclusion, the present study is the first comprehensive analysis of human HEV in Lithuania and the relationship between HEV and IBD. Moreover, it is the first study describing the genetic and kinetic properties of HEV strain isolated from a human patient in Lithuania. The results demonstrate that anti-HEV IgG and IgM prevalence rates in IBD patients and SOT recipients are higher compared to a healthy control group, consistent with similar findings from other European countries. The present study also identifies IBD, SOT, and age as risk factors associated with anti-HEV IgG presence. Moreover, phylogenetic analysis of locally isolated human HEV strain revealed a close genetic relationship with other Lithuanian HEV strains, isolated from wild animals and pigs, suggesting a possible HEV cross-infectivity between humans and animals in Lithuania.

## Figures and Tables

**Table 1:** Characteristics of studied and control groups.

Diagnosis	Sample Size (# Tested)	Age Range (Years)	Mean Age ± SD ^a^ (Years)	Gender (#M/#F ^b^)
Inflammatory bowel disease	203	18–80	40 ± 15	118/85
Ulcerative colitis	156	18–80	40 ± 15	89/67
W ^c^ anti-TNF treatment	29	18–57	34 ± 12	16/13
w/o ^d^ anti-TNF treatment	127	19–80	43 ± 16	73/54
Crohn’s disease	47	18–67	36 ± 13	29/18
w anti-TNF treatment	13	19–55	34 ± 12	9/4
w/o anti-TNF treatment	34	18–67	38 ± 14	20/14
Solid organ transplant recipients	63	19–76	52 ± 14	48/15
Liver allograft	5	21–54	43 ± 13	3/2
Kidney allograft	58	19–76	52 ± 14	45/13
Healthy control	100	20–85	53 ± 19	50/50
Total	366	18–85	46 ± 17	216/150

^a^ SD—standard deviation, ^b^ M/F—male/female, ^c^ w—with, ^d^ w/o—without.

**Table 2:** Seroprevalence of anti-HEV IgGs in healthy control and test groups.

	Healthy Control		Test Group Total	
	% IgG ^a^ Positive	(95%CI ^b^)	% IgG Positive	(95%CI)
Total	3.0	(0–6.3)	12.0	(8.1–15.9)
Age (years)				
<29	0		4.6	(0–9.6)
30–39	0		18.0	(7.4–28.7)
40–49	7.1	(0–20.6)	5.0	(0–10.5)
50–59	0		13.3	(3.4–23.3)
60–69	0		29.0	(13.1–45.0)
≥70	6.9	(0–16.1)	14.3	(0–32.6)
Gender				
Male	6.0	(0–12.6)	10.8	(6.1–15.6)
Female	0		14.0	(7.2–20.8)

^a^ IgG—immunoglobulin G, ^b^ CI—confidence interval.

**Table 3:** Seroprevalence of HEV (anti-HEV IgG, IgM, and IgG/IgM) by adult characteristics and clinical condition.

Diagnosis	% IgG ^a^ Positive (95%CI ^b^)	% IgM ^c^ Positive (95%CI)	% IgG/IgM Positive (95%CI)
Inflammatory bowel disease	12.3 (7.8–16.8)	6.4 (3.0–9.8)	3.0 (0.6–5.3)
Ulcerative colitis			
≤29	6.8 (0–14.3)	2.3 (0–6.7)	2.3 (0–6.7)
30–39	18.8 (5.2–32.3)	6.3 (0–14.6)	3.1 (0–9.2)
40–49	5.4 (0–12.7)	8.1 (0–16.9)	2.7 (0–7.9)
50–59	0	4.6 (0–13.3)	0
60–69	50.0 (23.8–76.2)	14.3 (0–32.6)	14.3 (0–32.6)
≥70	14.3 (0–40.2)	0	0
Crohn’s disease			
≤29	0	12.5 (0–28.7)	0
30–39	30.0 (1.6–58.4)	20.0 (0–44.8)	10.0 (0–28.6)
40–49	0	0	0
50–59	25.0 (0–67.4)	0	0
60–69	66.7 (13.3–100.0)	0	0
Solid organ transplant recipients	11.1 (3.4–18.9)	3.2 (0–7.5)	1.6 (0–4.7)
≤29	0	0	0
30–39	0	0	0
40–49	11.1 (0–31.6)	0	0
50–59	26.3 (6.5–16.1)	5.3 (0–15.3)	5.3 (0–15.3)
60–69	0	7.1 (0–20.6)	0

^a^ IgG—immunoglobulin G, ^b^ CI—confidence interval, ^c^ IgM—immunoglobulin M.

**Table 4:** Odds ratios of anti-HEV IgG positivity. Healthy control was used as a reference when calculating ORs in IBD and SOT recipient patients.

Factors	Anti-HEV ^a^ IgG ^b^	
	Odds Ratio (95% CI ^c^)	*p*
Age (years)	1.032 (1.007–1.057)	<0.01
Anti-TNF therapy	0	>0.05
Inflammatory bowel disease	4.541 (1.337–15.426)	<0.01
Ulcerative colitis	5.688 (1.633–19.817)	<0.01
Crohn’s disease	6.929 (1.628–29.487)	<0.01
Solid organ transplant received	4.042 (1.005–16.258)	<0.05
Kidney allograft	4.222 (0.979–18.213)	<0.05
Liver allograft	12.432 (0.992–155.757)	<0.05
Gender	0.958 (0.450–2.038)	>0.05

^a^ HEV—hepatitis E virus, ^b^ IgG—immunoglobulin G, ^c^ CI—confidence interval.

**Table 5:** Patients tested HEV RNA-positive.

No.	Patient ID	Age	Gender	Diagnosis	HEV ORF1 Positive	HEV ORF2 Positive	Anti-HEV ^a^ IgG ^b^ Positive	Anti-HEV IgM ^c^ Positive
1	BT1	22	Female	UC ^d^ with anti-TNF treatment	+	+	−	−
2	KA1	45	Female	Kidney allograft recipient	−	+	−	−
3	KA2	57	Male	Kidney allograft recipient	+	−	+	−
4	KA3	76	Male	Kidney allograft recipient	+	−	−	−
5	KA4	44	Male	Kidney allograft recipient	+	−	−	−

^a^ HEV—hepatitis E virus, ^b^ IgG—immunoglobulin G, ^c^ IgM—immunoglobulin M, ^d^ UC—ulcerative colitis.

## Data Availability

Partial gene sequence acquired during this study was submitted to GenBank (Accession number MT585816).

## Supplementary Materials

The following are available online at https://www.mdpi.com/article/10.3390/v13040670/s1, Figure S1: z-stacks of HEV infected MARC-145 cells captured every 1 μm (top to bottom; left to right). Scale bar: 50 μm.
