# Supplementary figures and images for: Molecular Characterization and Seroprevalence of Hepatitis E Virus in Inflammatory Bowel Disease Patients and Solid Organ Transplant Recipients
Source: Viruses. 2021 Apr 13;13(4):670. doi: 10.3390/v13040670 (PMC8070591; doi:10.3390/v13040670)

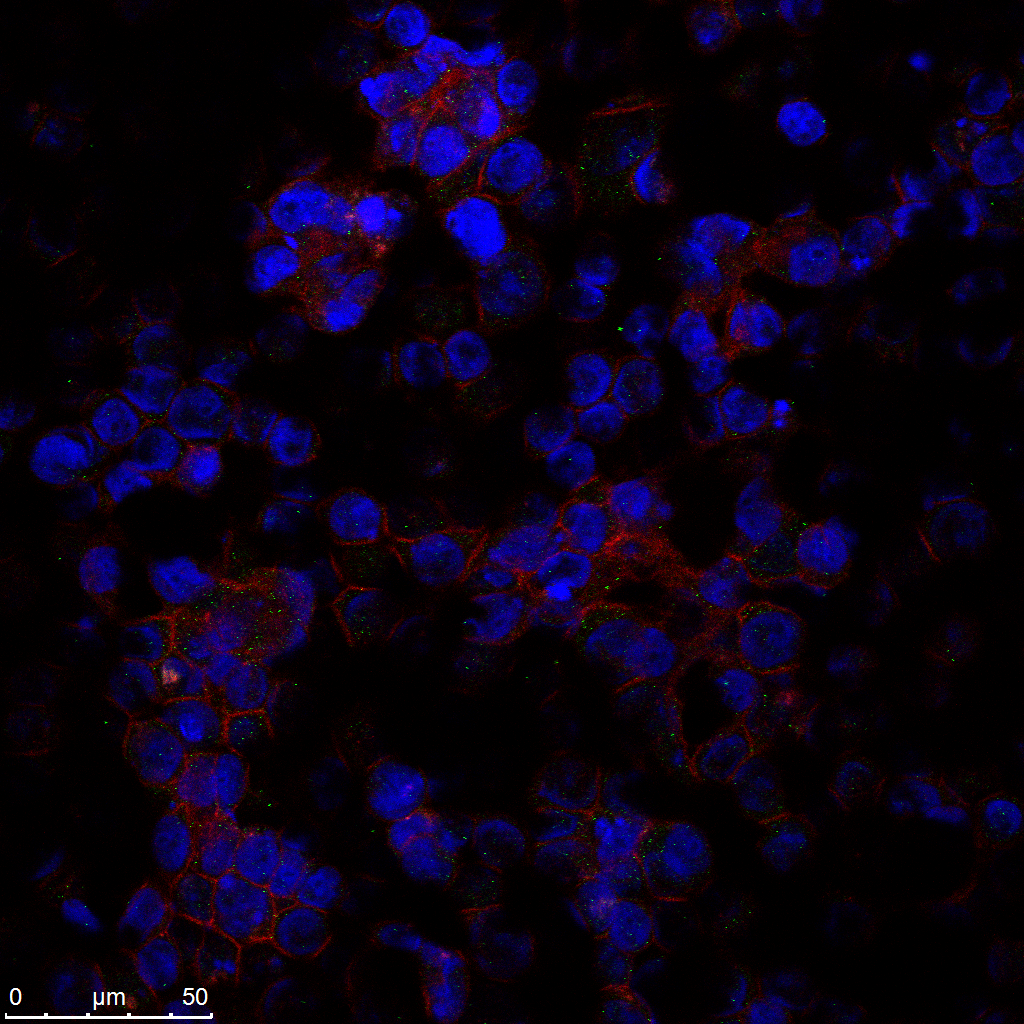

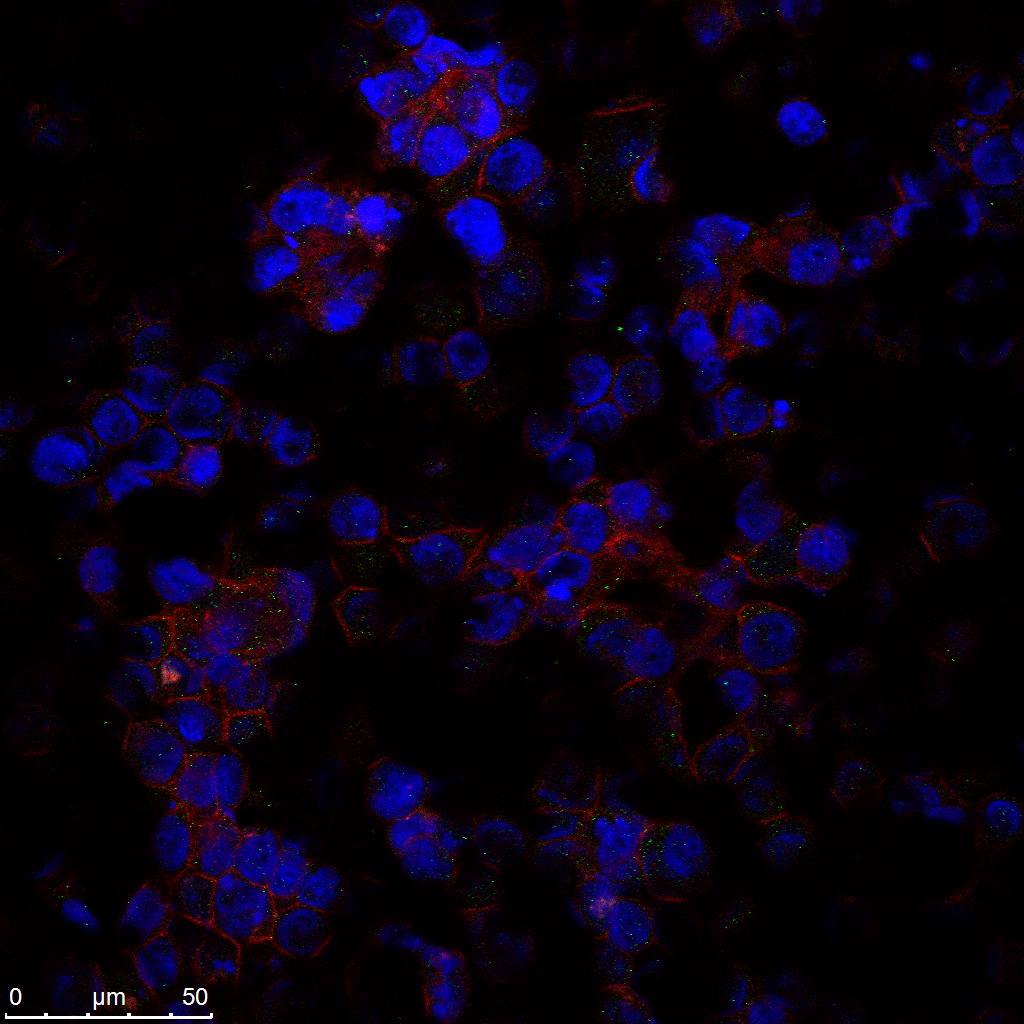

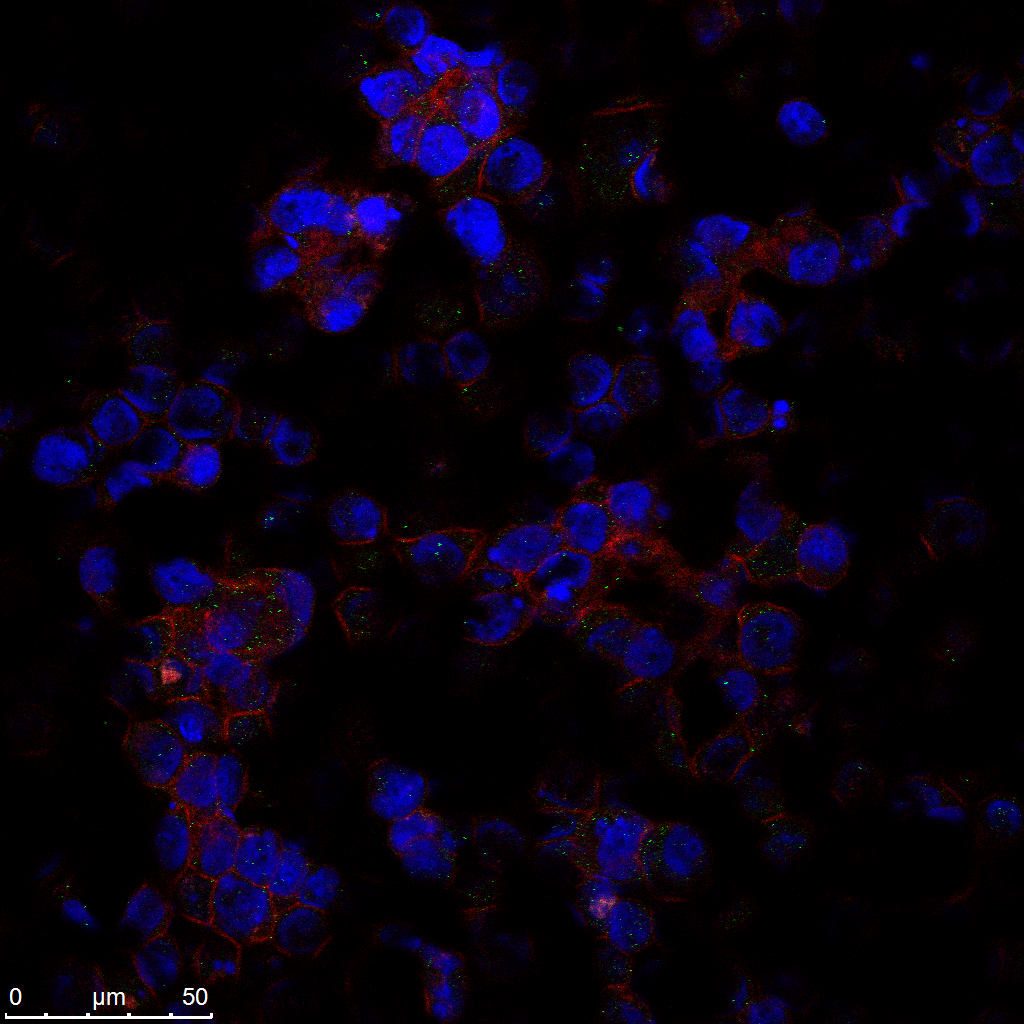

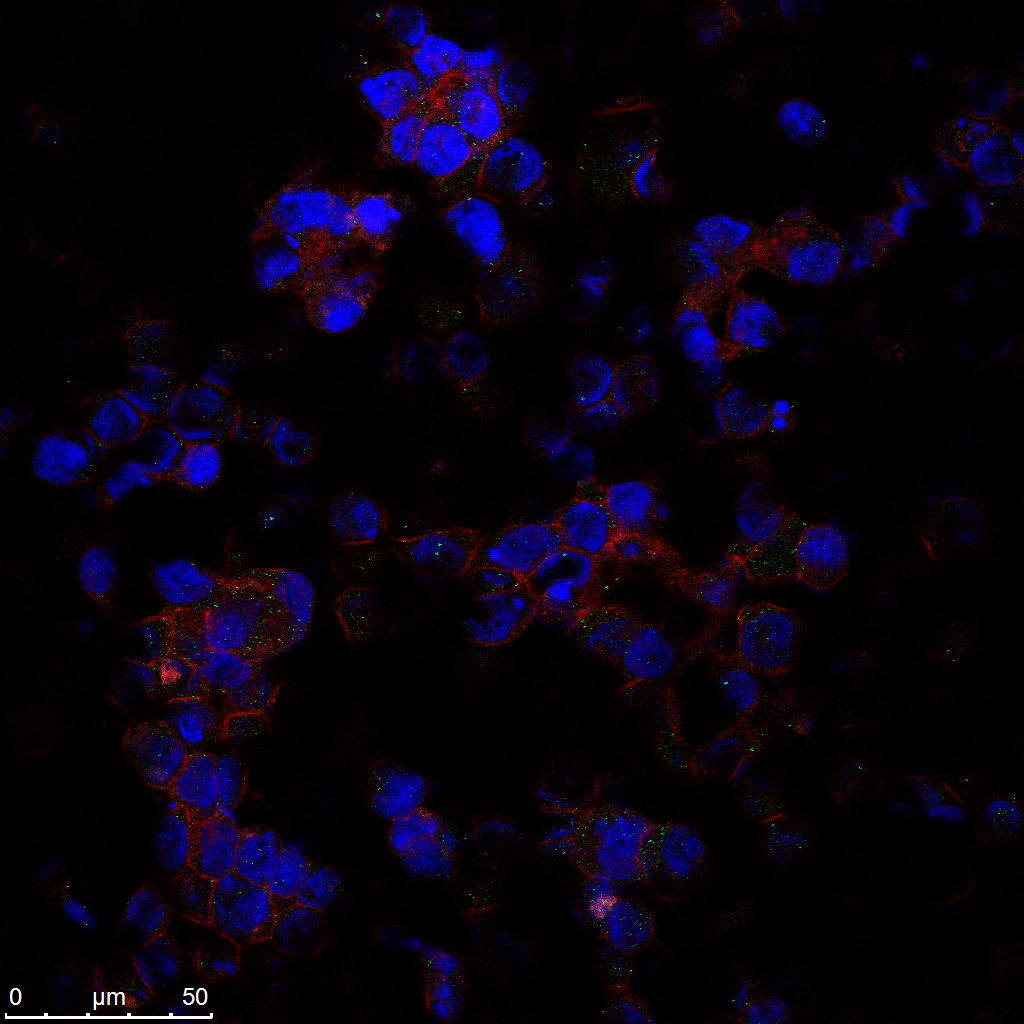

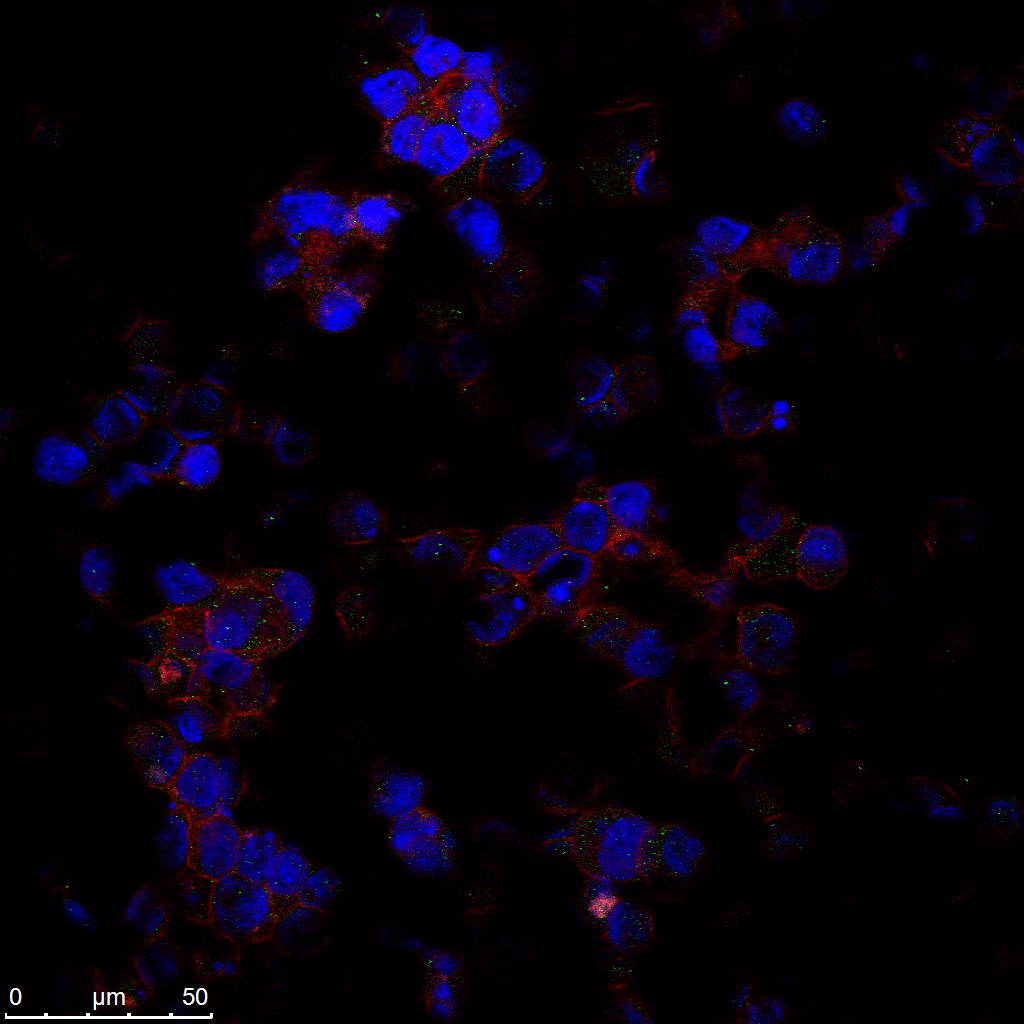

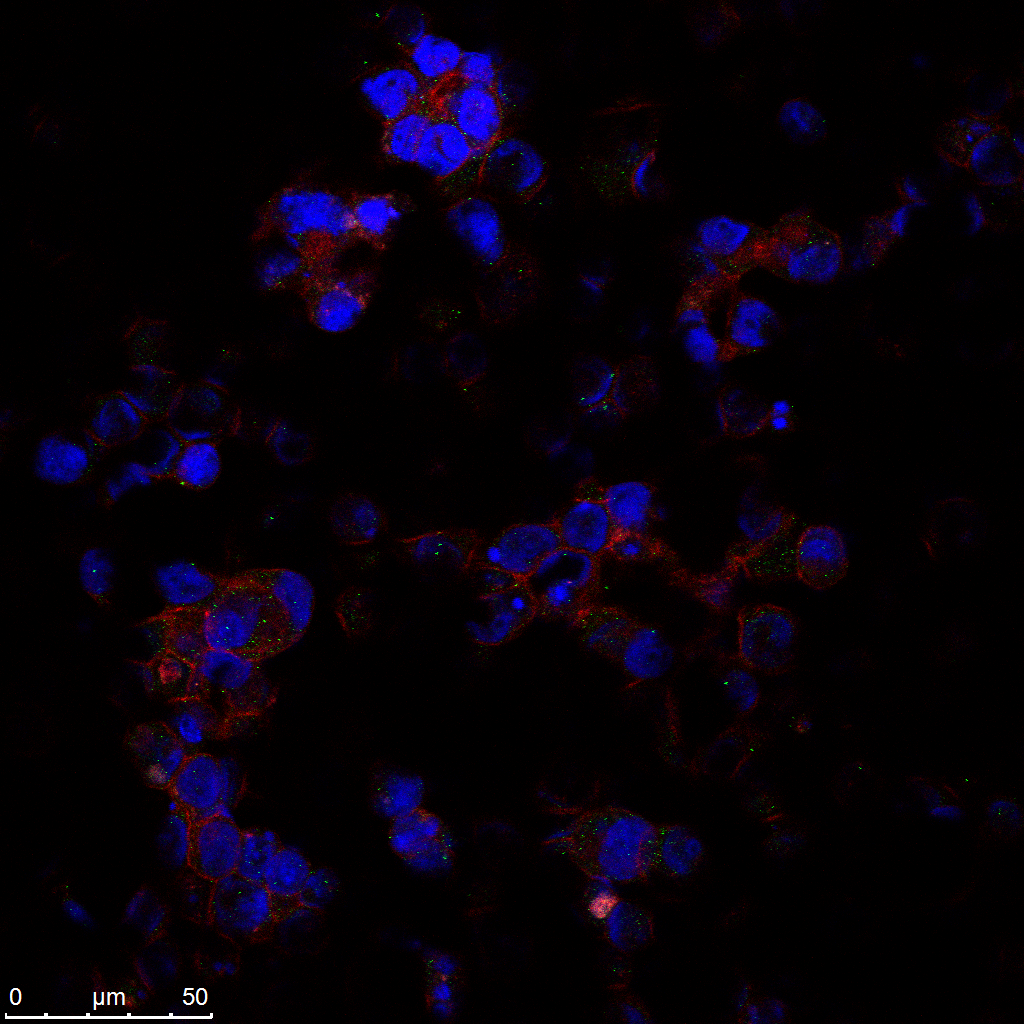

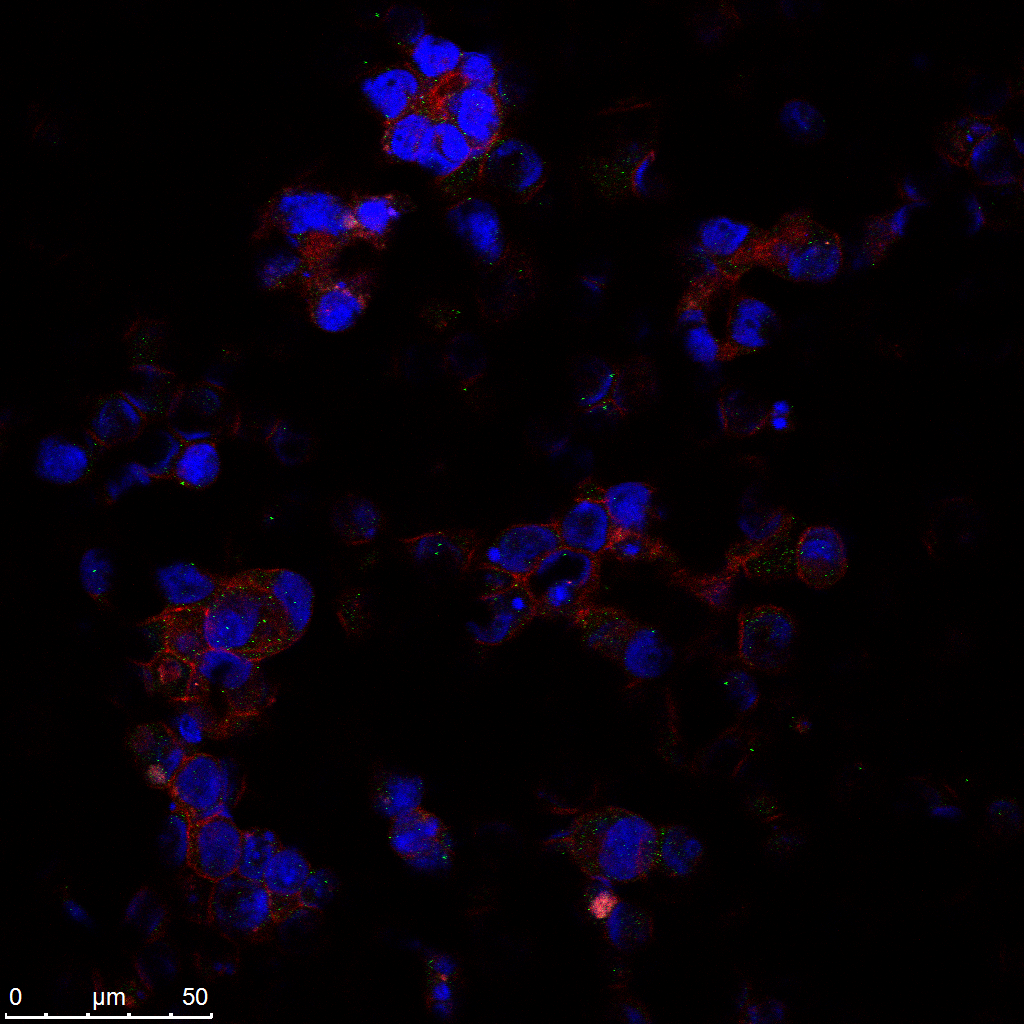

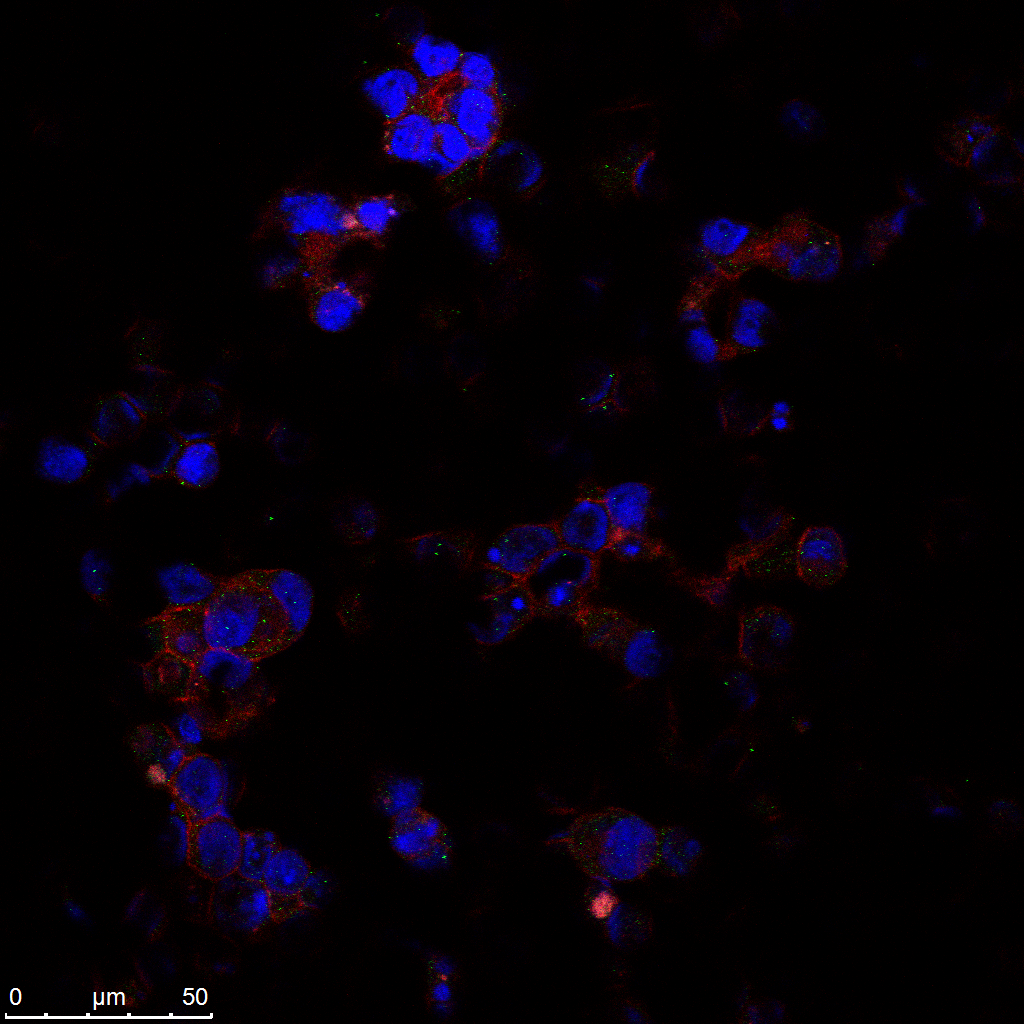

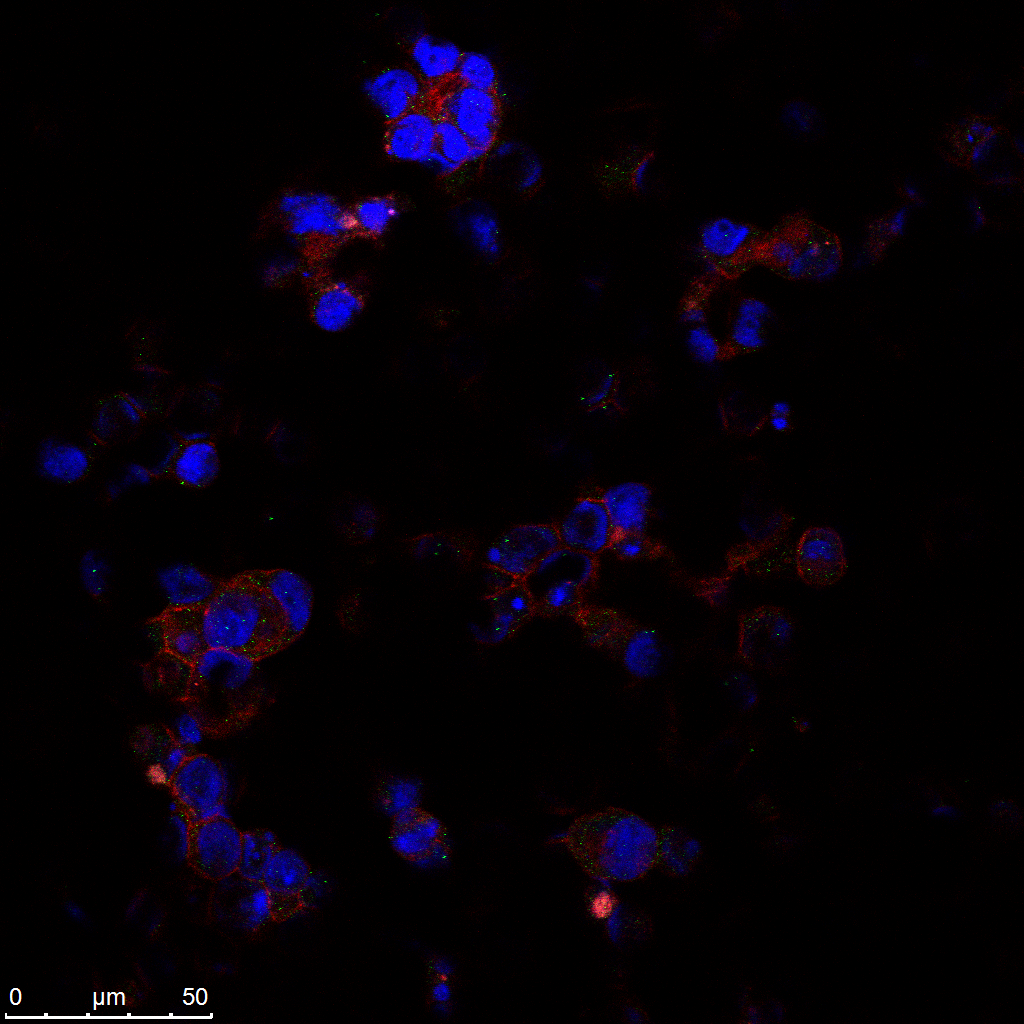

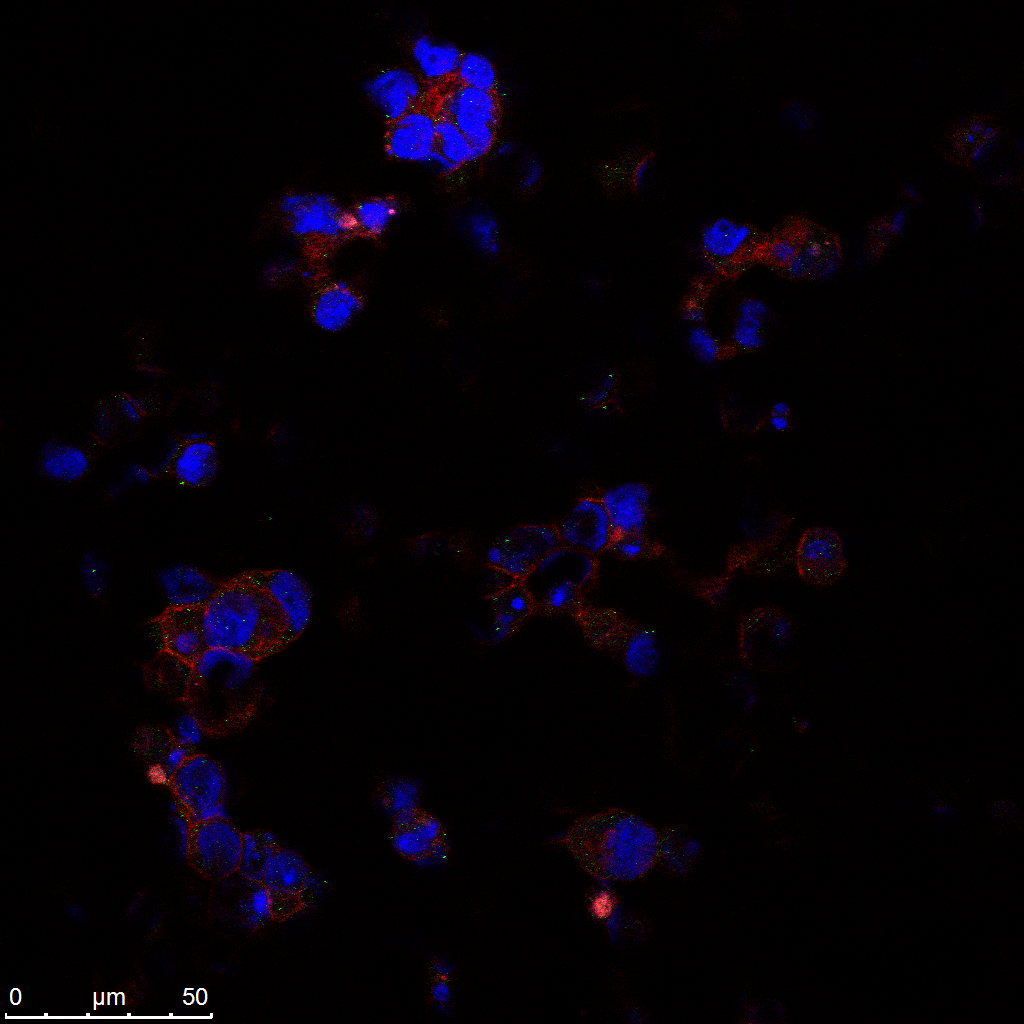

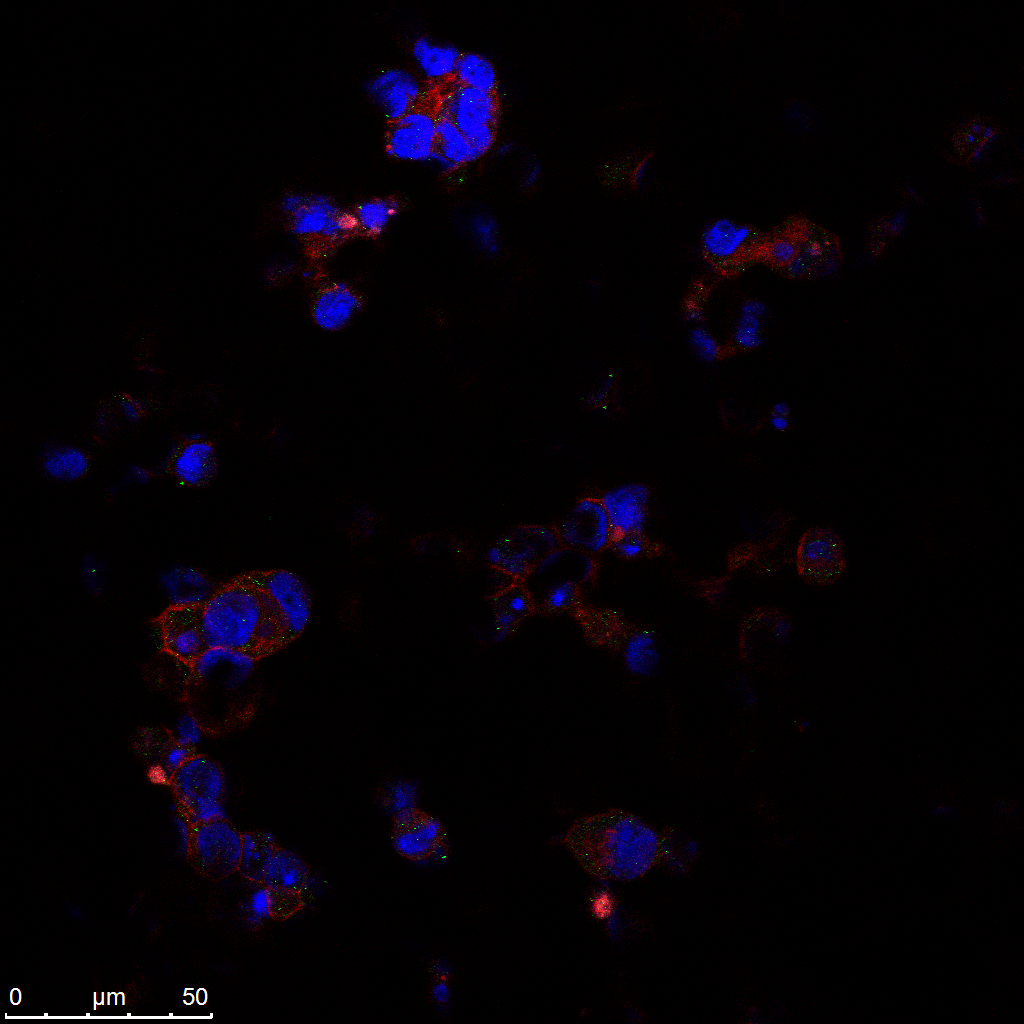

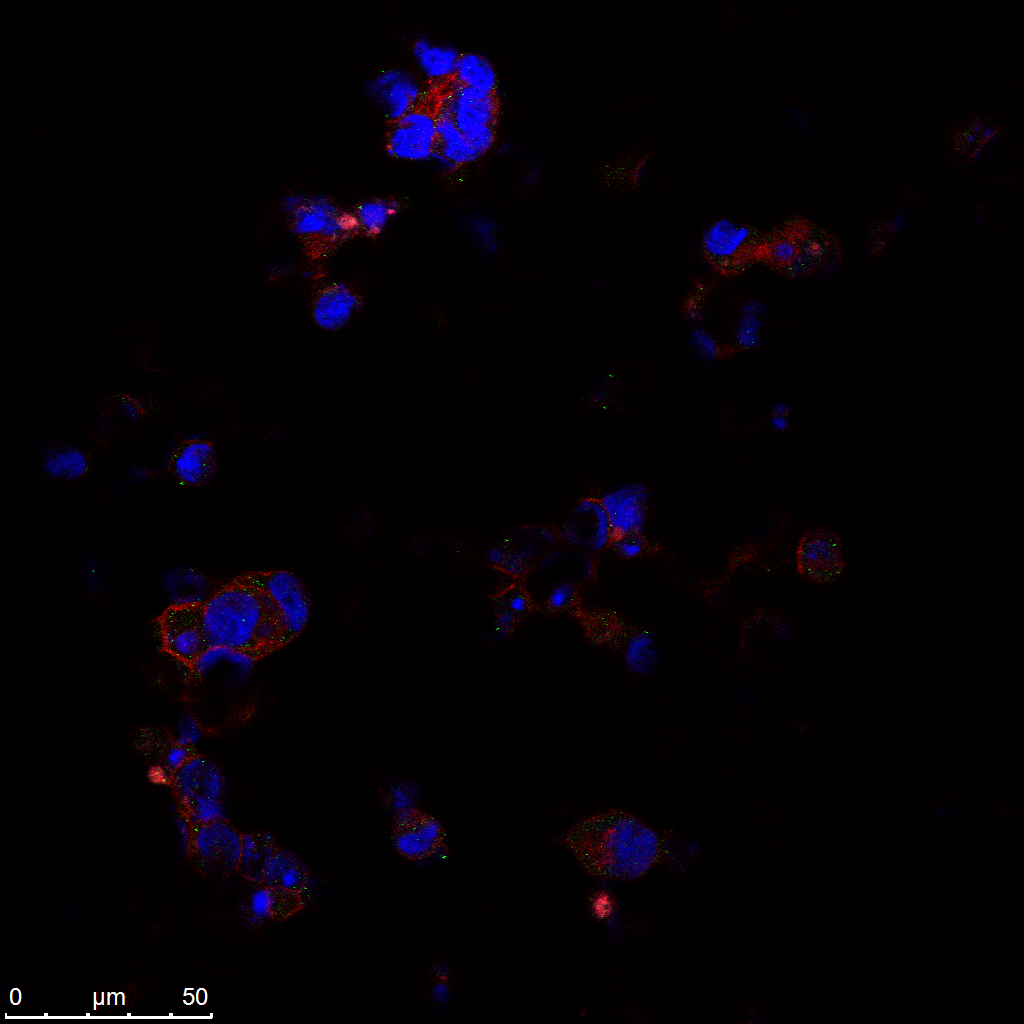

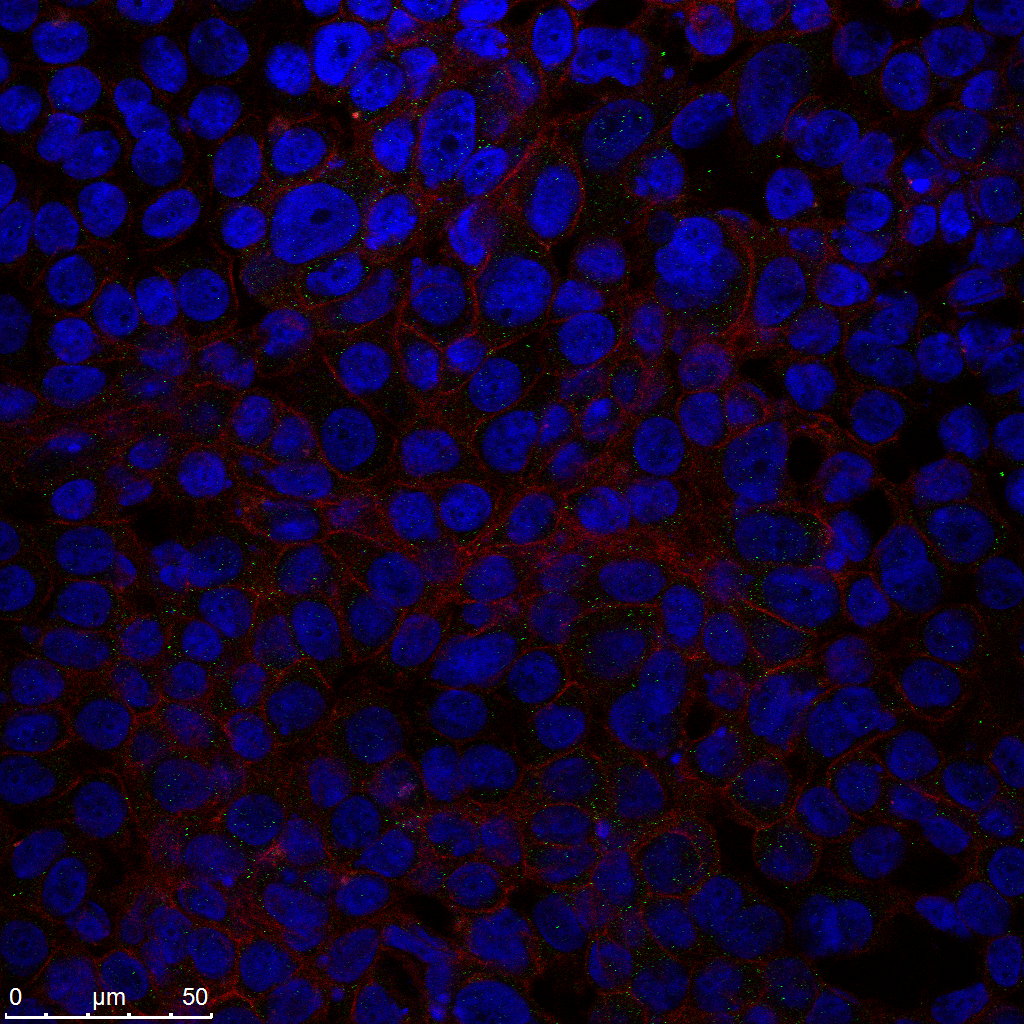

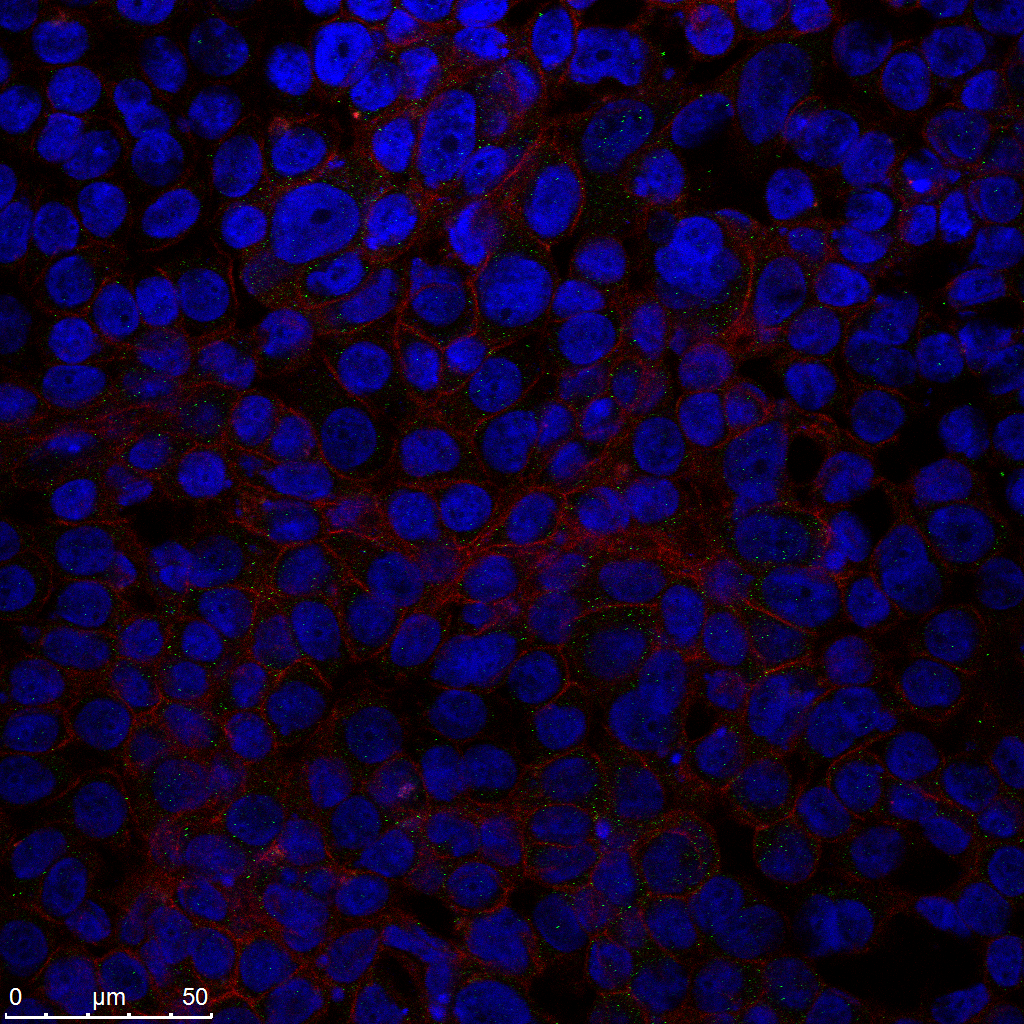

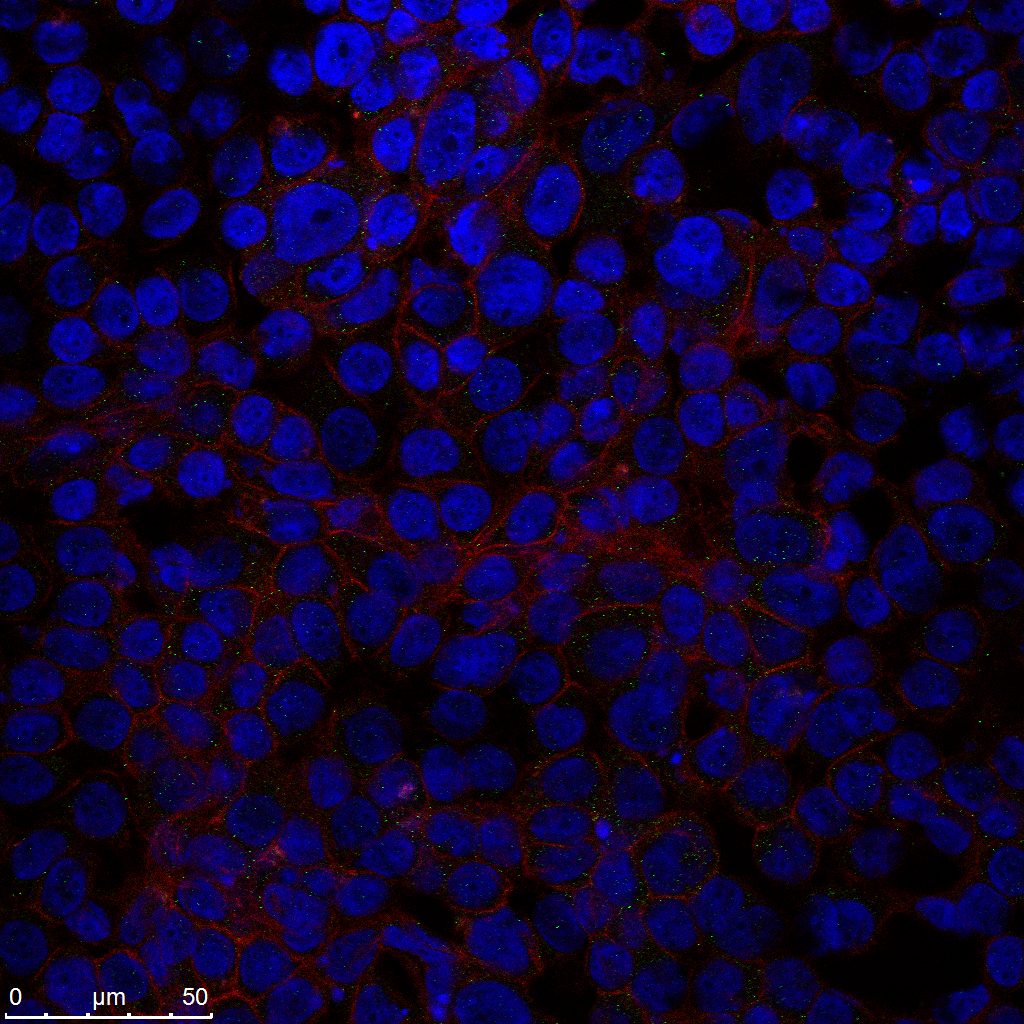

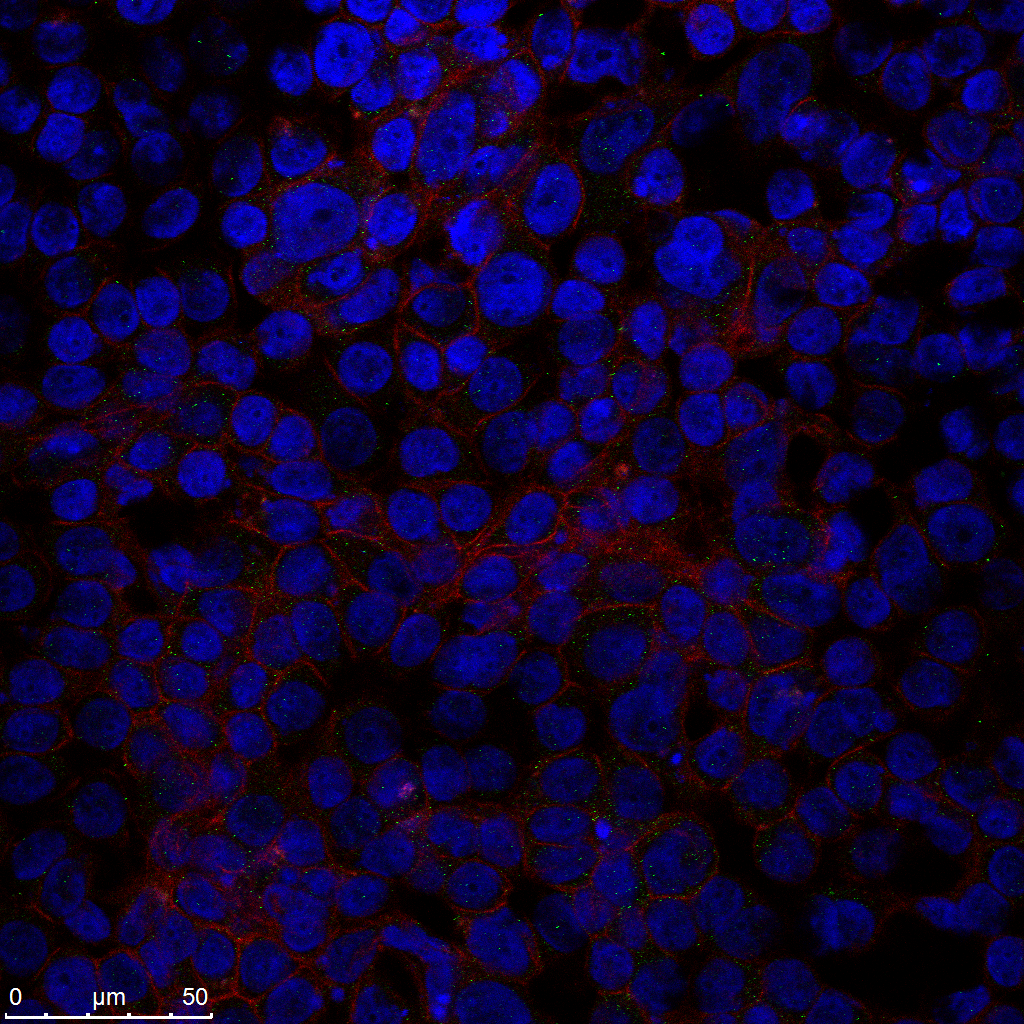

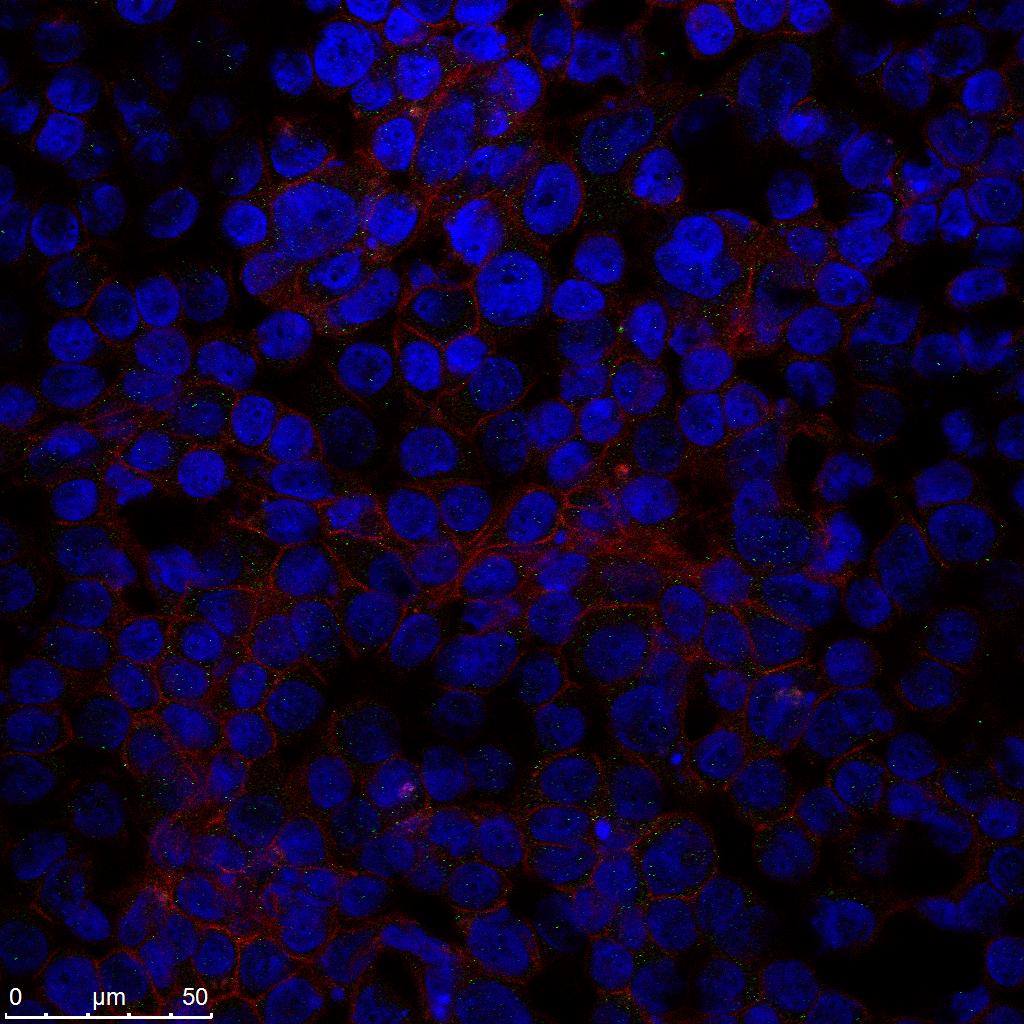

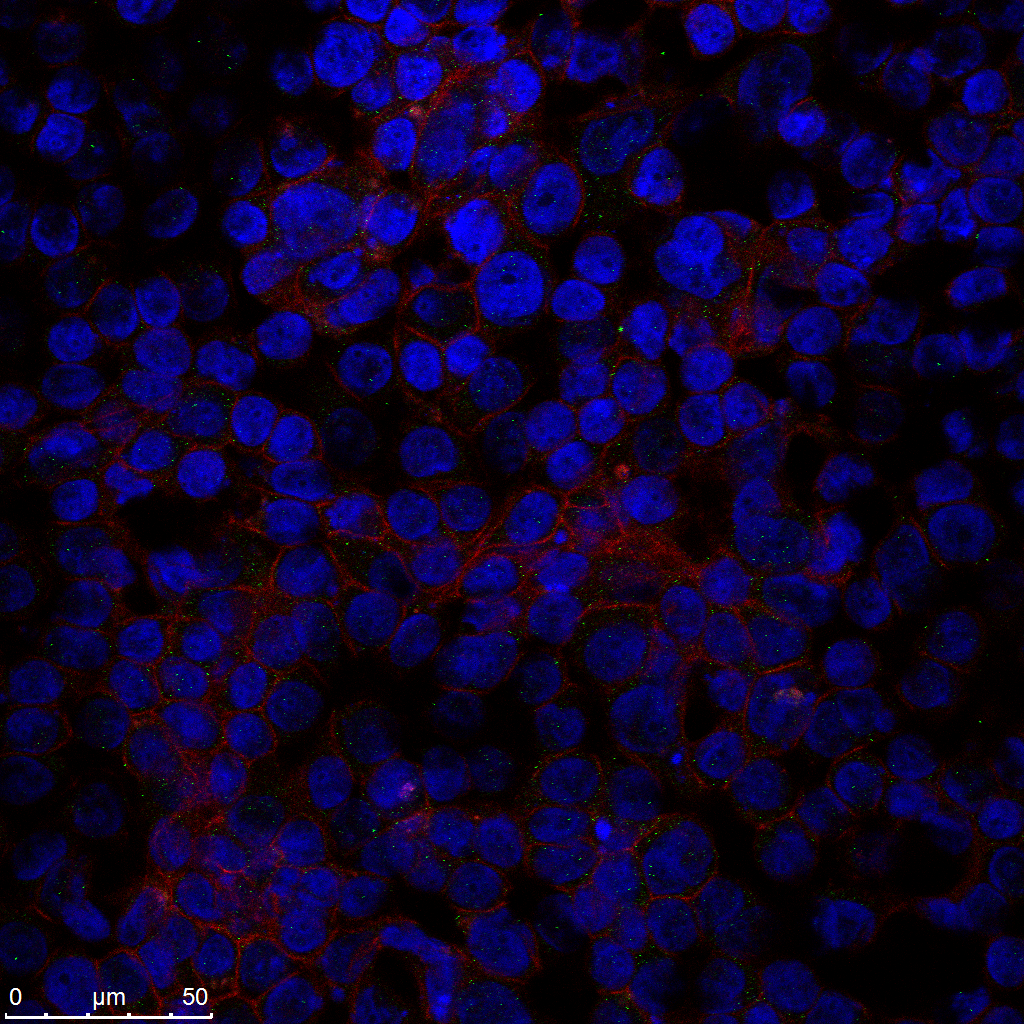

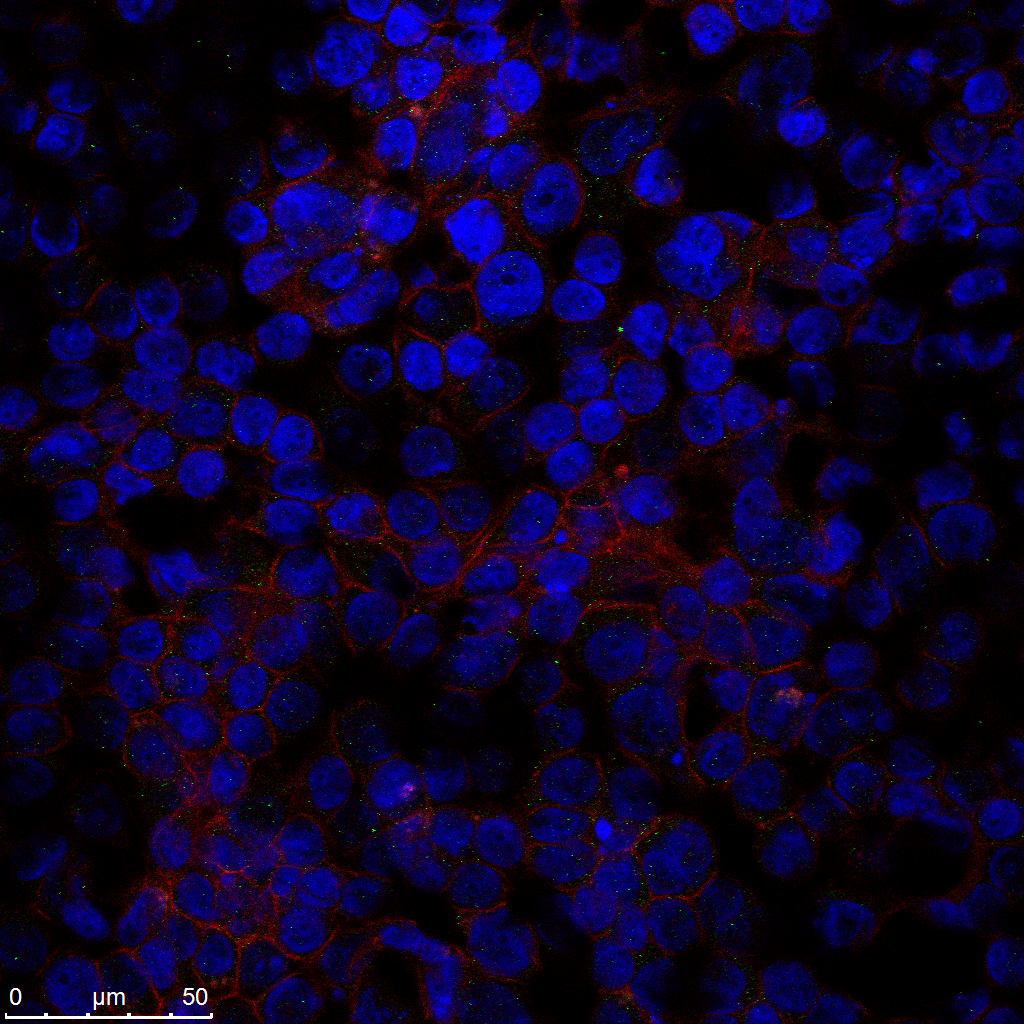

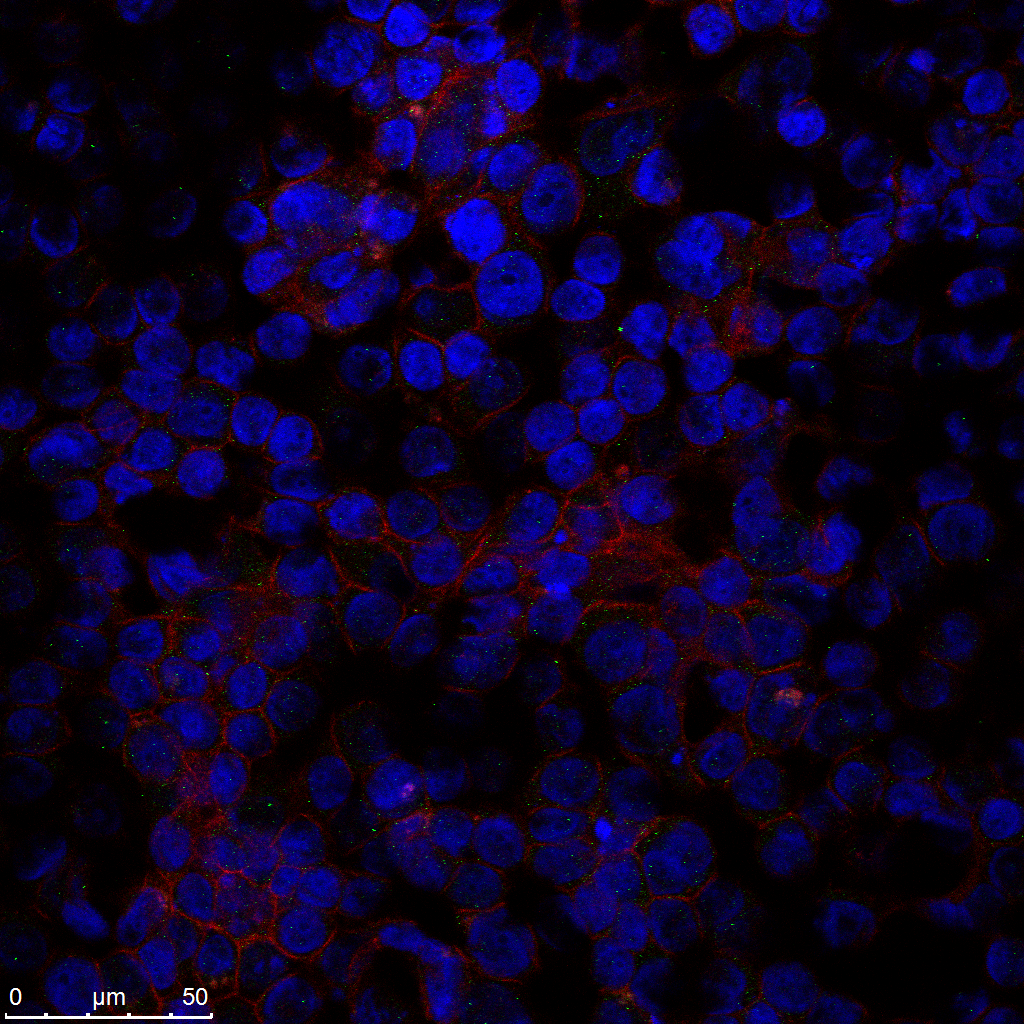

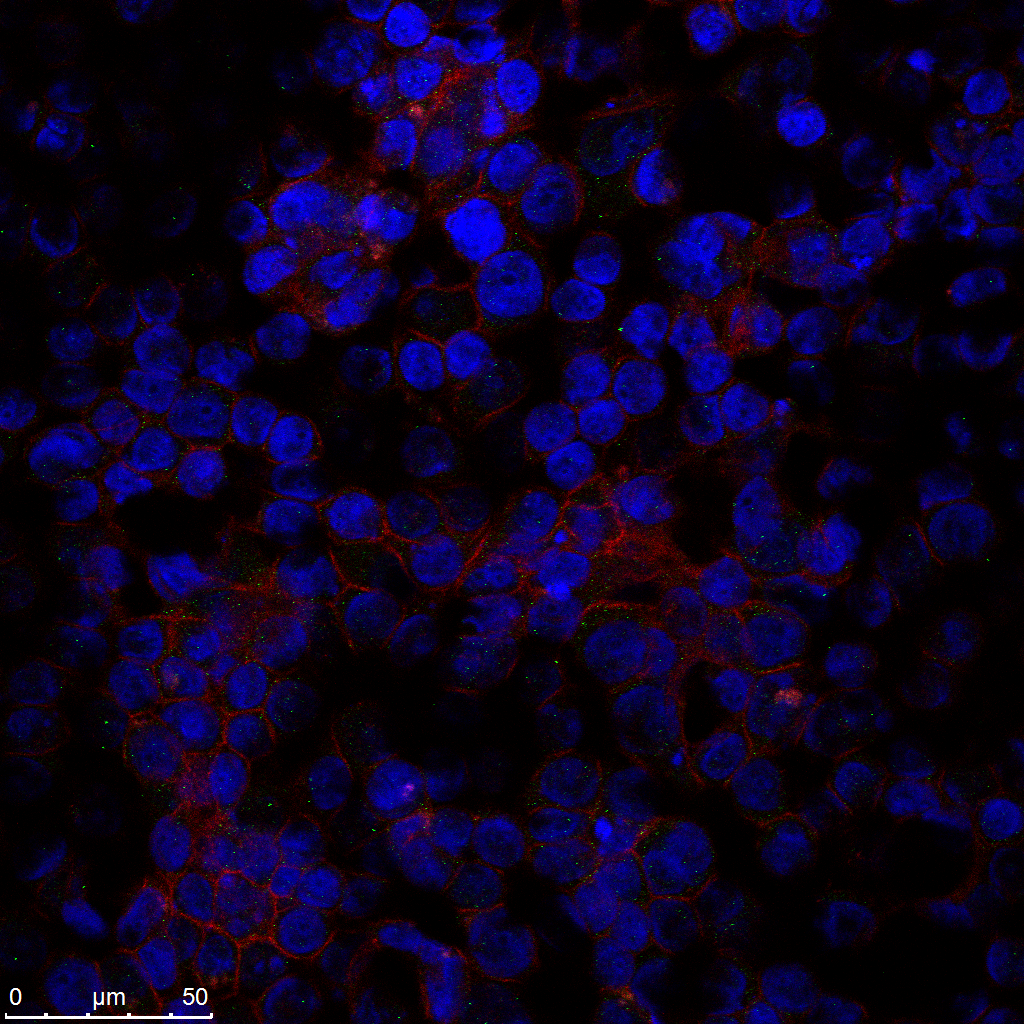

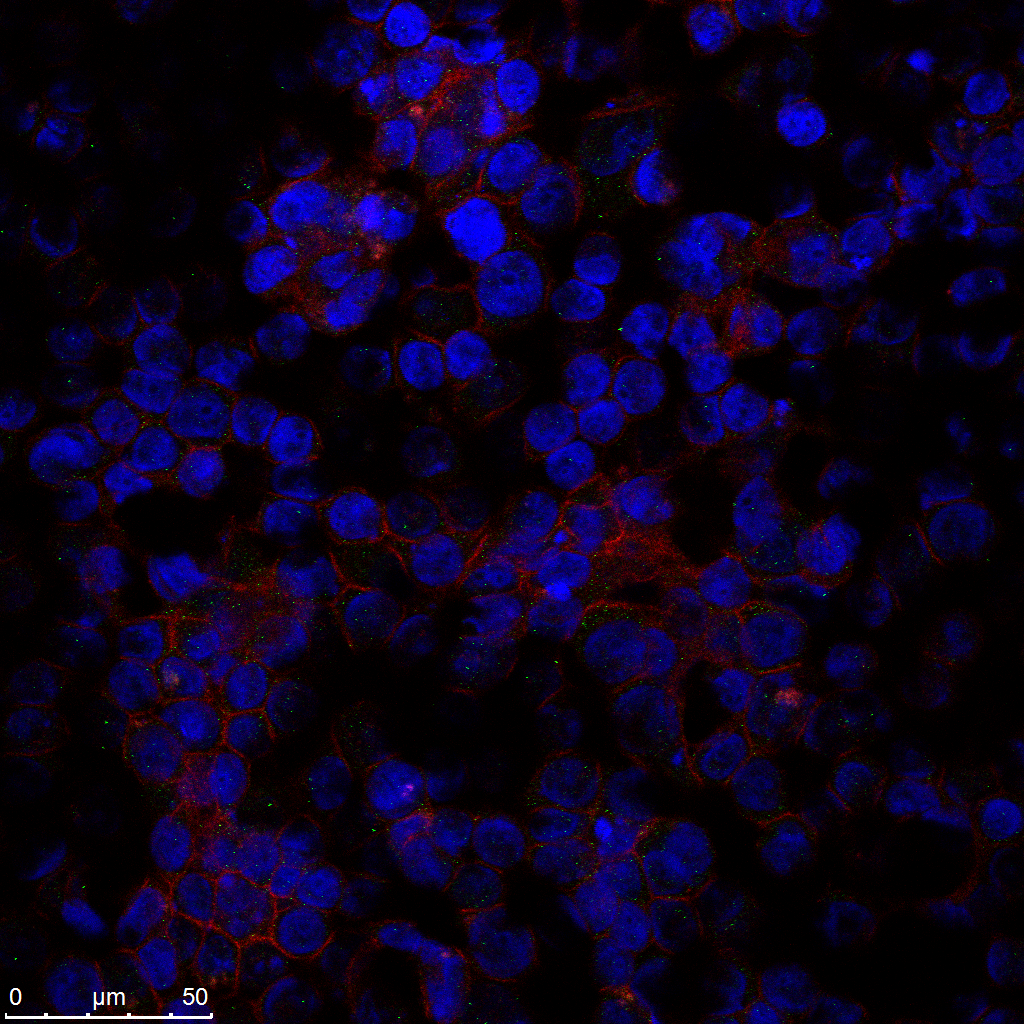

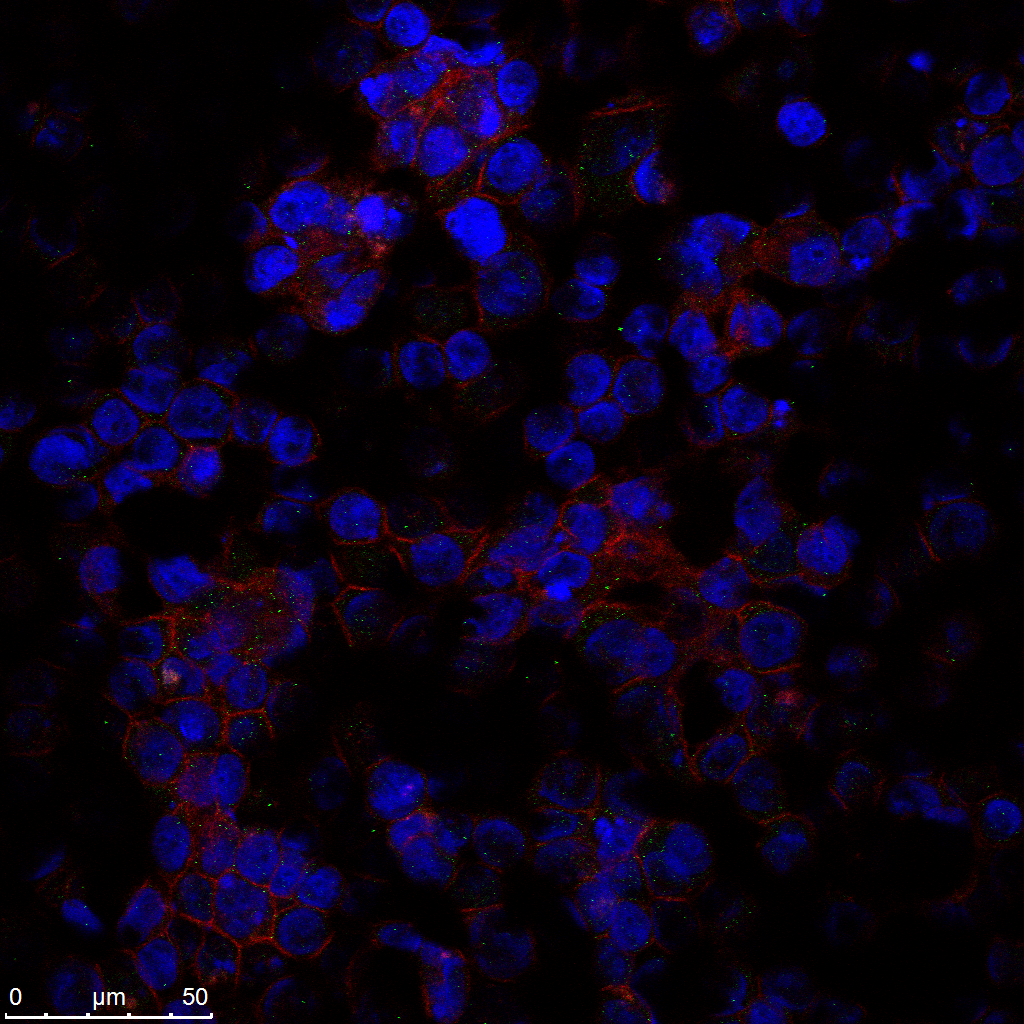

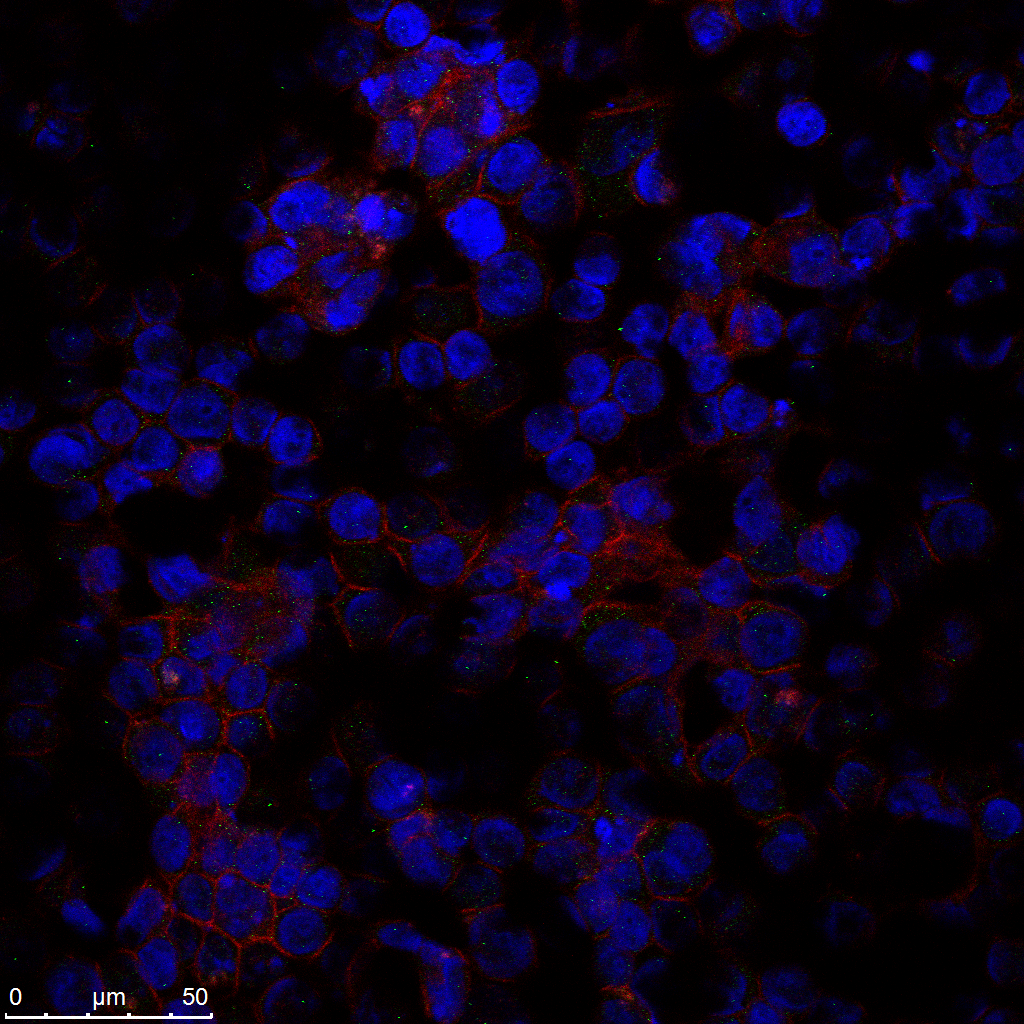

Supplement: Supplementary file 1 [file viruses-13-00670-s001.zip › Figure S1.docx]
